# A multimodal intervention to improve hand hygiene compliance via social cognitive influences among kindergarten teachers in China

**DOI:** 10.1371/journal.pone.0215824

**Published:** 2019-05-14

**Authors:** Xiaona Liu, Zhiguang Zhao, Wanli Hou, Suzanne Polinder, Ed F. van Beeck, Zhen Zhang, Yan Zhou, Gang Liu, Xu Xie, Jinquan Cheng, Jan Hendrik Richardus, Vicki Erasmus

**Affiliations:** 1 Department of Community Health and Health Services, Shenzhen Center for Disease Control and Prevention, Shenzhen, Guangdong Province, China; 2 Department of Public Health, Erasmus MC, University Medical Centre Rotterdam, Rotterdam, Zuid-Holland Province, The Netherlands; The University of Hong Kong, CHINA

## Abstract

Children attending kindergarten are at high risk for contracting infections, for which hand hygiene (HH) has been recognized as the most cost-effective prevention measure globally. Kindergarten teachers’ HH behavior plays a vital role in encouraging favorable hygiene techniques and environment. This study aims to evaluate the effectiveness of a multimodal intervention at changing kindergarten teachers’ HH behavior and social cognitive factors that influences HH behavior in China. The intervention named “Clean Hands, Happy Life” includes HH products with refills, reminders and cues for action, a kick-off event with awards, and training programs. We evaluated the intervention using a self-administrative questionnaire with a stratified random sample of 12 kindergartens. Two surveys was completed by 176 teachers at baseline and 185 after the 6-month intervention. Compared with the baseline scores, there was a significant improvement in the overall self-reported HH compliance of teachers (9.38 vs. 9.68 out of 10, *p* = 0.006), as well as teachers’ perceived disease susceptibility, disease severity and behavioral control after the intervention (*p*<0.05). We found that teachers’ HH compliance was likely to be higher among those who have better HH guideline awareness (β = 0.48, *p*<0.01) and perceived behavioral control (β = 0.26, *p* = 0.01), which explained 24.2% of the variance of self-reported compliance of teachers at baseline. The assessed intervention may provide Chinese kindergarten teachers with behavioral skills and cognitions that associated with the compliance of HH behavior. We thus recommend future intervention studies consider our HH behavior change techniques, address multiple social cognitive determinants of HH behavior and include the change of targeted influences in the impact evaluation.

## Introduction

Children attending kindergartens or child care centers are more susceptible to infectious diseases than children who are cared for in their own homes [1]. Children cared for at kindergartens have a 2–3 times greater risk of acquiring infections [2]. A recent study in Taiwan suggests that there were 136 laboratory-confirmed respiratory viral infections per 100 person years in the 2007 academic year among kindergarten attendees aged 2–5 years [3]. In addition, children attending kindergartens in China generally fall into the range of age between 3 and 6 years old–an age group that is highly susceptible to hand, foot and mouth disease (HFMD). A Chinese national surveillance registry documented over seven million probable HFMD cases between 2008 to 2012, of which 3.7% were laboratory confirmed and 0.03% were fatal [4].

Hand washing is regarded as a cost-effective and simple measure in the prevention of infectious diseases worldwide [5]. Previous studies showed that hand washing with soap reduced diarrheal diseases by 42–47% [6], and reduced the incidence of pneumonia among children under 5 years by 50% [7]. Furthermore, children normally develop their hygiene habits early in life if proper prompting and reinforcement are provided; teachers’ hygiene behavior plays a vital role in encouraging effective hand-washing techniques in order to curtail the spread of illness in the classroom [8]. Practicing good hand hygiene among teachers has been recognized as an important measure that decreases the risk of becoming infected with the viruses that cause HFMD at schools [9], and is regulated by China’s national guideline on public health response for HFMD prevention and outbreak in schools, including kindergartens [10]. Despite the proven importance of hand washing, compliance with hand-washing guidelines is generally suboptimal among children’s care givers in both resource-rich and resource-limited areas. Only 17% of children’s care givers washed their hands with soap after using the toilet according to a study in 11 African countries [11]. Several interventions aiming at increasing compliance with hand-washing guidelines have shown varying levels of effectiveness [12].

Previous research on hand washing in China has generally focused on the determinants of hand hygiene (HH) among healthcare workers to prevent healthcare-associated infections [13,14]. An exception is a survey using a representative sample of Chinese adults aged 18–60 years including healthcare workers and teachers. The authors found that the hand-washing behavior of Chinese adults was strongly associated with level of urbanization of area of residence, level of education and level of knowledge of HH [15]. However, a review of hand-hygiene improvement strategies concluded that addressing only factors such as knowledge and awareness is not enough to change HH behavior, and combinations of different determinants showed better results [12]. Little is known about the social cognitive influences that underlie the hand-washing behavior of kindergarten teachers in China.

As a starting point to investigate hand-washing behavior among Chinese kindergarten teachers, we used social cognitive constructs that were developed for a previous study among child day care centers in the Netherlands [16], derived from the Health Belief Model. The model suggests that people’s engagement (or lack of engagement) in health-promoting behavior can be explained by their perceptions of personal susceptibility to a particular illness, perceptions of severity of a particular illness, perceived behavioral control/self-efficacy, balanced against perceived benefits of action and barriers to action [17]. The Health Belief Model has gained substantial empirical support since its development in the 1950s, and is particularly notable given the diverse populations, health conditions, and health-related behaviors examined and the various study designs [17]. Theoretically, the application of a chosen behavioral change strategy as part of the HH improvement intervention will alter specific social cognitive determinants, which in turn will change HH behavior [18].

We hypothesize that our multimodal behavioral change strategies (which together make up the intervention titled “Clean Hands, Happy Life”) developed based on the factors identified from the behavior change theory and evidence from Dutch day care centers will be effective at changing HH associates and eventually improving HH compliance of kindergarten teachers in China. The purpose of the present study is to assess the social cognitive factors that influence the hand-washing behavior of kindergarten teachers in China; to evaluate the effectiveness of our theory- and evidence-based intervention at changing hand-washing behavior, as well as the determinants thereof using self-reported HH compliance as the outcome measure. Our intervention has lately proven to be effective in decreasing HFMD [19], this evaluation thus will also enable us to gain insights on possible reasons behind the success of behavioral change techniques to prevention infections in kindergartens.

## Materials and methods

### Study design and participants

**Fig 1:**
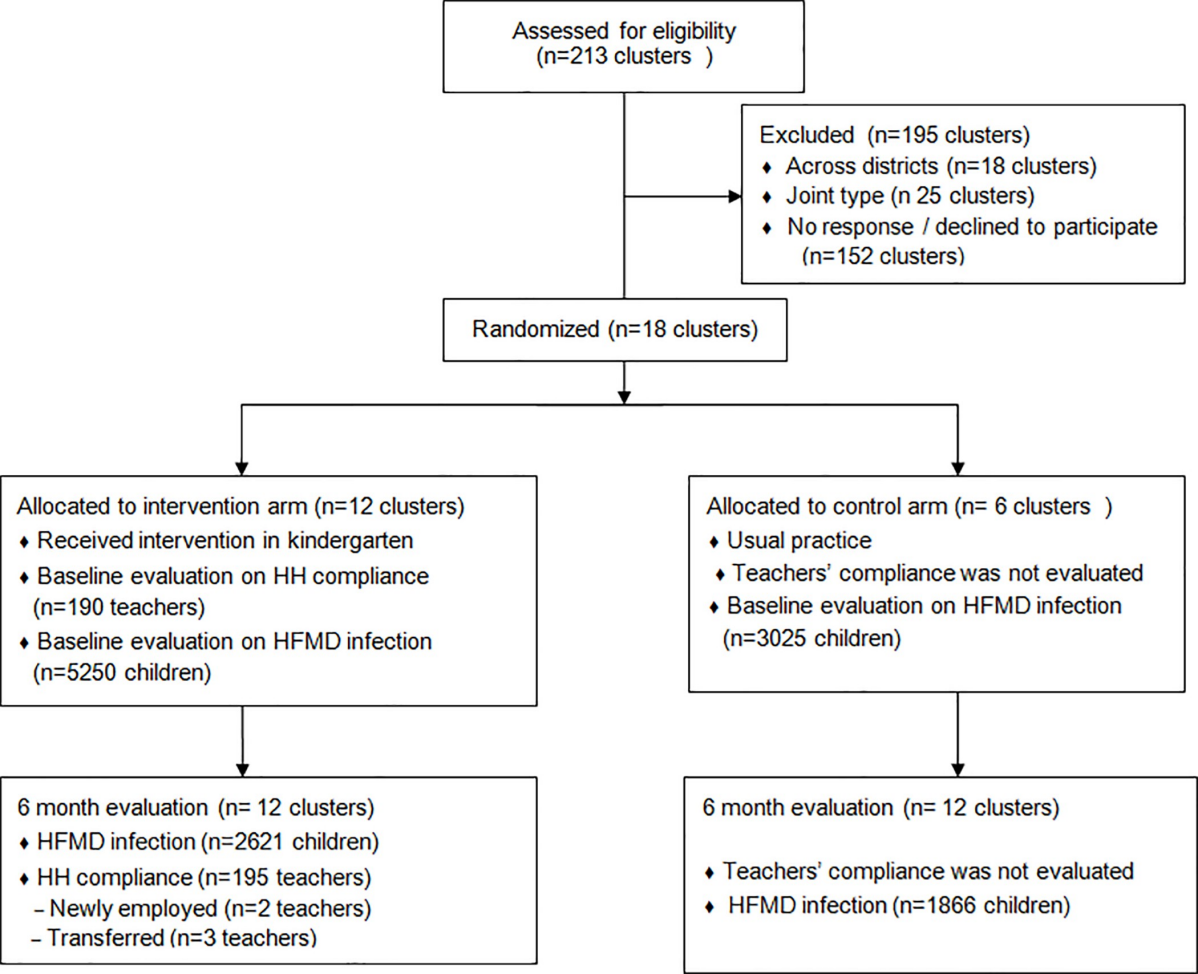
Flow diagram for cluster randomized trial evaluating the effect of a hand hygiene intervention on self-reported hand hygiene (HH) compliance of kindergarten teachers to decrease hand, foot and mouth disease (HFMD) in Baoan and Nanshan district, Shenzhen. Note: This sub study of the trial focuses on evaluating HH compliance of kindergarten teachers.

This is a before-and-after study among kindergarten teachers and is a part of a cluster-randomized controlled trial among children attending kindergartens conducted in Shenzhen between May and October, 2015 (see Fig 1). A multimodal intervention was conducted in a stratified random sample of 12 kindergartens that equally represent 3 types of kindergartens (i.e. government-owned, privately-owned and migrant–a type of privately owned kindergarten specifically targeting children of domestic migrants) and 2 types of areas (i.e. urban and semi-urban) in Shenzhen, China. All teachers who were mainly responsible for classrooms receiving the intervention were invited to fill in a self-administrative and anonymous questionnaire, after giving informed consent. After 6 months the survey was repeated at the 12 kindergartens, however, the respondents before and after the intervention might not be completely the same, as there were changes regarding new and transferred employment of working staff within kindergartens during the follow-up. We calculated the sample size of the trial using methods recommend by Breukelen and colleagues [20], taking into account the intra-cluster correlation coefficient of kindergartens, the cost per cluster and cost per person, the expected effect on behavior change, the power and the significance level of the study. Our process of randomization and calculating sample size have been previously reported [19]. This trial’s protocol is registered and approved by the Ethics Committee of Shenzhen CDC (Registration no. 2015005) and the Netherlands Trial Registry (TC-5395). Informed consent to participate was collected from the teachers.

### Intervention program

This intervention was known as “Clean Hands, Happy Life洁净双手,快乐生活” in China, and consisted of four components. First, the following HH products were provided free of charge with refills for six months: soap dispensers, towel dispensers, paper towels and soap. Second, reminders and cues for action were provided free of charge, including posters, stickers, HH-related reading books, memory games, coloring pages and a hand-washing diploma designed for children. Third, one kick-off event was performed in each kindergarten with four stations, including a hand hygiene e-game with tablets, hand-washing instructions, educational story time, and diploma awards. Fourth, training was given to educate kindergarten teachers about the national guideline of HH, illustrating why (reasons), when (moments) and how (steps) to perform hand washing. This included a hand-washing exercise using UV Glow Cream and an information booklet outlining the content of the training, which was based on an HH training session developed in Dutch day care centers [21]. All intervention materials were pre-tested to be appropriate to read or play for the Han Chinese (ethnicity) children from mainland China.

### Outcome measurement

The teachers were asked to complete a questionnaire before and after the intervention on self-reported HH compliance and social cognitive determinants of hand-washing behavior. The questionnaire also included sociodemographic questions including age, gender, occupation duration, education level and whether or not the teacher was living with children under 14 years old. There were also HH-related illness history questions including whether or not they were suffering from dry hands or eczema.

The questionnaire was based on an HH questionnaire tested among care givers in Dutch day care centers [16]. Self-reported HH compliance was assessed by calculating the mean of 13 questions, with the questions resembling the specific activities for which hand washing was indicated in the Chinese kindergarten guidelines. Teachers were asked to answer these questions giving an answer ranging from 0 (never) to 10 (always). The following 5 assessed social cognitive determinants were derived from the Health Belief Model [17]: awareness of the guidelines, knowledge of the guidelines, perceived disease susceptibility (i.e. perception of the risk of contracting an infection in kindergartens), perceived disease severity (i.e. beliefs regarding the seriousness of contracting an infection in kindergartens), and perceived behavioral control (i.e. perceived difficulty or ease of performing hand washing). Table 1 lists the assessed psychometric properties with example questions, answer options and internal consistency measured with Cronbach’s α. Constructs of the psychometric properties were finalized until Cronbach’s α ≥ 0.70 [22]. Guideline knowledge was measured with 2 true/false questions and guideline awareness was measured on a 7-point Likert scale, while the rest of the social cognitive determinants were measured on a scale from 0 to 10.

**Table 1:** Example questions and scale reliability with means for assessment of social cognitive determinants of hand hygiene (HH) behavior of kindergarten teachers before and after the “Clean Hands, Happy Life” intervention in Shenzhen, China.

Covariates	No. of items	Example question	Answer options	Cronbach’s α	Before		After		*p*-value
					Mean	IQR	Mean	IQR	
Awareness of the guidelines	1	I know exactly what the rules are for washing hands.	Certainly not (1)–certainly yes (7)	N/A	6.58	1.00	6.73	0	0.077
Knowledge of the guidelines	2	Hands should be washed before preparing for lunch	False (0), true (1)	0.888	0.98	0	0.99	0	0.558
Perceived disease susceptibility	2	If your colleagues do not wash their hands, what is the chance that this will cause a child in your class to become infected?	Very small (0)–very big (10)	0.958	1.51	2.00	2.16	4.00	0.030*
Perceived disease severity	2	How severe are the possible consequences for a child when he/she contracts an infection?	Not server (0)–very sever (10)	0.932	7.30	4.50	8.16	2.75	0.011*
Perceived behavioral control	5	To what extent are you sure that you will be able to wash your hands when you are busy?	Completely unsure (0)–completely sure (10)	0.727	8.72	1.35	9.09	1.00	0.001*

Note: IQR, Interquartile range IQR = Q_3_-Q_1_

* denotes significant at P < 0.05.

All covariates involve repeated measurements at the kindergartens received the intervention; Wilcoxon signed-rank tests were used to compare the repeated measurements after clustering the data by kindergartens.

### Data analyses

Data were analyzed using SPSS version 24.0 (SPSS Inc., USA). Descriptive analyses were used to describe personal characteristics of participants. The mean and interquartile deviation (IQD) were calculated as statistical measures to describe all ordinal variables, including social cognitive determinants of HH behavior and HH compliance in different activities. The IQD represents the distance between the 25th and 75th percentiles of scores. Both t-tests and chi-square tests were used to compare personal sociodemographic characteristics before and after the intervention; Nonparametric Wilcoxon signed-rank tests were performed to compare repeated measurements of social cognitive association and HH compliance changes before and after the intervention at kindergartens, after aggregating results at the kindergarten level. Multilevel univariable and multivariable linear regression analyses were performed to assess sociodemographic and social cognitive covariates of the overall HH compliance for all cases at baseline corrected for clustering of the data within kindergartens. A *P*-value of less than 0.05 was considered significant and only variables with significance were tested in multivariable analyses. Multilevel univariable analyses were also conducted on the measured covariates after the intervention with adjustment for clustering all data by kindergartens. Finally, possible differences in effect of the intervention and significant covariates in the multivariable model at the different time points was examined by including relevant two-way interactions. All assumptions underlying the regression models, such as normality of the residuals and multicollinearity, were assessed graphically.

## Results

A total of 361 teachers completed the survey, with 176 during the baseline period (response rate: 92.6%) and 185 in the post-intervention period after implementing 6 months of the intervention (response rate: 94.9%). Table 2 shows the comparison of baseline respondents’ characteristics and post-intervention respondents and demonstrates that with regard to the social demographics and HH-related illness history of respondents, there was no significant difference between before the intervention and after the intervention (*p*>0.05).

**Table 2:** Comparison of respondents’ characteristics before (N = 176) and after the intervention (N = 185).

	Before	After		
	*n* (%)	*n* (%)	χ^2^	*p*-value
Gender				
Women	167 (94.8)	174 (94.1)	0.002	0.967
Men	2 (1.1)	2 (1.1)		
Age	29.1 ± 8.66	28.9 ± 8.67	0.226	0.821
Number of years working as a teacher	7.09 ± 7.03	5.94 ± 5.60	1.723	0.086
Education level				
High school or lower	51 (29.0)	64 (34.6)	1.347	0.510
Junior college	81 (46.0)	77 (41.6)		
College or higher	44 (25.0)	44 (23.8)		
Living with children under 14 years old	74 (42.0)	75 (40.5)	1.015	0.602
Suffer from dry hands				
Sometimes / always	138 (78.4)	149 (80.5)	0.251	0.616
Never	38 (21.6)	36 (19.5)		
Suffer from eczema				
Sometimes / always	22 (12.5)	26 (14.1)	0.189	0.664
Never	154 (87.5)	159 (85.9)		

Note: Chi-square test and t-test were used for comparing characteristics measured as categorical and continuous variables, respectively.

### Effect of the intervention on specific and overall HH compliance

Table 3 displays that the overall mean self-reported compliance with HH guidelines was 9.68 (scale, 0–10) after the intervention, which is significantly higher than the overall mean 9.38 at baseline (*P* = 0.006). More specifically, after the intervention kindergarten teachers reported significantly more hand washing behavior than baseline after coughing in their hands and/or sneezing, after blowing their nose, after changing a diaper with feces, after contacting bodily fluids and soiled textiles, after going to the toilet, after wiping the nose of a child, after wiping a child’s bottom, and before helping a child with food (*p*<0.05). Little variation was found among self-reported HH compliance in specific activities after the intervention (IDQ = 0), with the exception of hand washing after coughing in the hands and/or sneezing (IQR = 1).

**Table 3:** Effect of the “Clean Hands, Happy Life” intervention on hand hygiene compliance of kindergarten teachers per type of activity on a scale of 0 (never) to 10 (always).

Type of activity	Before		After		
	Mean	IQR	Mean	IQR	*p-*value
Before the preparation of the lunch	9.49	0	9.68	0	0.156
Before peeling of fruit	9.47	0	9.73	0	0.374
After coughing in the hands and / or sneezing	8.98	1	9.33	1	0.084 *
After blowing your nose	9.05	1	9.56	0	0.006 *
After changing a diaper with feces	9.40	0	9.46	0	0.004 *
After contacting with body fluids (saliva, vomit, blood, wound, urine, snot)	9.65	0	9.88	0	0.007 *
After playing outside	9.32	0	9.54	0	0.720
After contacting soiled textiles (dirty washcloths, bibs, burp cloths, towels)	9.24	1	9.72	0	0.000 *
After going to the toilet	9.61	0	9.87	0	0.034 *
Before you go eat yourself	9.48	0	9.79	0	0.093
Before you help a child with food	9.34	0	9.68	0	0.020 *
After wiping the nose of a child	9.28	0	9.68	0	0.028 *
After wiping a child’s butt	9.68	0	9.90	0	0.005 *
**Overall HH compliance**	9.38	0.47	9.68	0.23	0.006 *

Note

*indicates *P*<0.05

IQR indicates interquartile range, IQR = Q_3_-Q_1_; All data were clustered by kindergartens. Wilcoxon signed-rank tests were used to compare the repeated measurements at kindergarten level.

### Effect of the intervention on social cognitive influences

Table 1 shows the means and IQR of scores of the social cognitive covariates before and after the intervention. Scores for guideline awareness, guideline knowledge, perceived disease severity and behavioral control were above mid-scale, whereas the scores for perceived disease susceptibility were below mid-scale. This suggests that the respondents reported good self-awareness of the HH guidelines (mean>6, scale:1–7) and knowledge of hand-washing behavior (mean>0.9, scale:0–1), perceived kindergarten infection to be serious (mean>7, scale: 0–10) and had great confidence in HH compliance (mean>9, scale:0–10). However, they believed that the chance of getting infections in kindergarten was small (mean<3, scale: 0–10). Compared with baseline scores, perceived disease susceptibility, disease severity and behavioral control were significantly increased after the intervention (*p*<0.05). However, teachers’ awareness and knowledge of HH compliance were not significantly changed (*p*>0.05) (Table 4).

**Table 4:** Social and cognitive determinants of hand hygiene compliance of kindergarten teachers before and after the “Clean Hands, Happy Life” intervention in Shenzhen, China.

Covariates	Before				After		
	Univariate		Multivariable	(R^2^ = 0.242)	Univariate		Multivariable^#^
	β-Coefficient (95%CI)	*p*-Value	β-Coefficient (95%CI)	*p*-Value	β-Coefficient (95%CI)	*p*-Value	*p-*Value
Gender	0.63(-0.14–2.80)	0.568			0.18(-1.2–1.56)	0.794	
Age	0.02(-0.01–0.04)	0.225			0.01(-0.1–0.02)	0.344	
Number of years working as a teacher	0.01(-0.03–0.04)	0.741			0.01(-0.02–0.04)	0.384	
Education level	0.03(-0.31–0.25)	0.836			0.00(-0.19–0.18)	0.971	
Living with children under 14 years old	0.47(0.02–0.92)	0.042*	0.62(0.22–1.02)	0.003*	0.33(-1.58–2.24)	0.737	0.775
Suffer from dry hands	-0.38(-0.92–0.17)	0.176			0.01(-0.34–0.37)	0.943	
Suffer from eczema	0.82(0.15–1.49)	0.017*	0.80(0.19–1.42)	0.011*	-0.03(-0.45–0.39)	0.891	0.839
Awareness of the guidelines	0.65(0.42–0.87)	<0.01*	0.48(0.23–0.73)	<0.01*	0.20(-0.03–0.43)	0.082	0.361
Knowledge of the guidelines	-0.32(-2.33–1.7)	0.760			-0.36(-2.1–1.35)	0.681	
Perceived disease susceptibility	0.08(-0.02–0.17)	0.110			0.03(-0.01–0.08)	0.151	
Perceived disease severity	0.07(0–0.13)	0.036*	0.03(-0.03–0.91)	0.269	0.04(-0.01–0.09)	0.159	
Perceived behavioral control	0.45(0.28–0.63)	<0.01*	0.26(0.05–0.47)	0.014*	0.12(0.27–2.23)	0.136	0.001*

Note

*indicates *P* <0.05

All coefficient were calculated with adjustment for clustering by kindergartens.

^#^ This multivariable model included two-way interaction of follow-up time and the factors significantly associated with the hand hygiene compliance before the intervention, and adjustment for clustering by kindergartens. *P*-value obtained in this model examines the interaction for evidence of intervention effect modification.

### Determinants of HH compliance of teachers

Table 4 demonstrates the determinants of hand hygiene compliance of kindergarten teachers before and after the intervention. Univariate linear regression analysis suggests that the overall self-reported HH compliance of teachers at baseline was significantly associated with their awareness of the guidelines (β = 0.65, *p*<0.01), perceived disease severity (β = 0.07, *p* = 0.04) and behavioral control (β = 0.45, *p*<0.01). In addition, teachers who were living with children under 14 years old (β = 0.47, *p* = 0.04) and had suffered from eczema in the past (β = 0.82, *p* = 0.02) were more likely to be compliant with the guidelines. Corrected for living with children under 14 years old, a history of eczema and perceived disease severity, the HH compliance was significantly associated with guideline awareness (β = 0.48, *p*<0.01) and perceived behavioral control (β = 0.26, *p* = 0.01) in the multivariable model, which explained 24.2% of the variance of self-reported HH compliance at baseline. The overall model fit was x^2^/df = 1.56; RMSEA = 0.04. None of the identified covariates were found to be significantly different between before and after the intervention (*p*>0.05), except the perceived behavioral control was statistically improved after the intervention (*p* = 0.001), after correcting for interactions of other identified covariates and follow-up time (the model fit: x^2^/ df = 1.03; adjusted R^2^ = 0.18; RMSEA = 0.06).

## Discussion

This study reveals that there were significant improvements in self-reported HH compliance and in the cognitive determinants–namely, perceived disease susceptibility, severity and behavioral control–for kindergarten teachers after 6-month implementation of the intervention “Clean Hands, Happy Life” at their workplace.

The intervention increased the HH compliance of teachers significantly but slightly, due to the very good compliance reported at baseline. A lower baseline compliance may allow the similar intervention to create a larger effect as what we previously found in a Dutch study [16,23]. The improved self-reported HH compliance might be explained partially by the improved perceived control we identified, and could be better explained by including other social cognitive factors that were not directly measured in this study. For example, a recent study in Zimbabwe suggests that frequent self-reported hand washing was associated with performing a more effective hand-washing technique, which was mainly influenced by the availability of hand-washing stations and action planning [24]. Our previous study in child day care centers showed that self-reported HH compliance was also associated with habit [16]. However, we could not measure the association between the improved compliance and the improved disease susceptibility and severity due to the change of respondents after the intervention. According to the Health Belief Model, perceived disease susceptibility and severity could function as perceived benefits to taking or planning action [25]. If an individual believes that a particular action will reduce susceptibility to an infection or decrease its seriousness, then he or she is likely to engage in that behavior regardless of objective facts regarding the effectiveness of the action. More studies are necessary to dynamically investigate the influence of social cognitive factors on the change of HH behavior.

The respondents reported very good knowledge and awareness of the HH guideline before the intervention. As a consequence, we did not observe a statistically significant increase in knowledge and awareness after the intervention. On the other hand, this confirms the widely acknowledged notion that good knowledge or awareness alone may not be essential to improve healthy behavior [26]. When exploring the social cognitive determinants of self-reported HH compliance, we found that living with children under 14 years old, suffering from eczema, guideline awareness and perceived behavioral control were significantly associated with HH compliance before the intervention; however, none of the identified factors were associated with the compliance after the intervention. This confirms the finding of a previous study on an HH awareness-raising campaign that essentially changing behavior requires more than knowledge and an emphasis on the importance of washing hands [27]. In addition, this is in line with our previous study in child day care centers in the Netherlands, which also found that having children at home, guideline awareness and perceived behavioral control were associated with self-reported HH compliance [16]. Number of children at home was the only social covariates of HH compliance that we identified in this study. We believe that it serves as an effective cue or trigger for kindergarten teachers to believe they are at risk of infections and eventually to take preventive actions.

To the best of our knowledge, this is the first study evaluating the effects of a large-sale HH intervention on both self-reported behavior and its underlying social cognitive influences in China’s kindergartens. Although there were personnel changes during the 6-month follow-up, no significant differences were found between respondents before and after the intervention. Additionally, this is a part of a larger study on evaluating our developed intervention targeted to decrease HFMD among children attending kindergartens in China. The findings in this study contribute to explain why our intervention was effective at reducing HFMD infections [19].

Our study has several limitations. The reliance on self-reported compliance to elucidate actual compliance is a prospective one, as self-reported HH behavior is likely to be an overestimation of actual behavior uptake because of social desirability bias. However, due to the nature of this study, it was impossible to measure actual HH practices in the recruited kindergartens with regard to the intervention. Although we had measured the utilization of HH products in addition to self-reported HH behavior, it’s difficult to tell the difference in usage between teachers and children, since all HH facilitates and products were shared by children and teachers in the intervention arm. Also, before the intervention the respondents reported very good knowledge and awareness. This did not allow us to obtain significant improvement in knowledge and awareness satistically after the intervention. Furthermore, our questionnaire was previously validated in Dutch day care settings, and might reflect actual behavior and psychological status insufficiently in Chinese kindergarten [28]. Another limitation is that we did not find any social cognitive factors that were significantly associated with the self-reported compliance after the intervention, despite the fact that the explained variance of the multivariable model before the intervention was relatively high (24.2%). Additionally, we used the before-and-after cross-sectional analysis of the intervention, which does not imply causality. We did not follow up the respondents at the baseline individually due to the cluster-randomized nature of the trial and the anonymous nature of the survey, thus the findings may suffer from conjunction fallacy in probability judgment [29]. Finally, our sample may not be representative of all teachers in kindergartens globally, and we cannot exclude effects regarding order or repetition of questions in the survey.

Hand washing has proven to be an effective measure to reduce the incidence of diarrhea and respiratory infections in kindergartens [30]. The “Clean Hands, Happy Life” intervention increased kindergarten teachers’ estimation of the severity of the infections and risk of being infected due to lack of compliance with HH guidelines, as well as increased their perception of their competence to be compliant. However, it is not easy to change deep-rooted behaviors like hand washing. Future health education and health-promotion interventions should be based on specific social cognitive and environmental determinants for HH compliance, in particular physical and cognitive barriers to practicing hand washing for kindergarten teachers. Moreover, impact evaluations of further interventions should take into account the change of targeted covariates, as well as process factors that are critical to successful implementation and to a long-term impact. Observing and explaining the HH behavior of teachers with additional social cognitive and environmental factors in a randomized controlled trial with repeated measures will be our next step in the Chinese kindergartens.

## Supporting information

S1 File Protocol of the “clean hands happy life” study. (DOCX)

S2 File The CONSORT 2010 checklist. (DOC)

S3 File Hand hygiene questionnaire for teachers_CN. (DOCX)

S4 File Hand hygiene questionnaire for teachers_EN. (DOCX)

S5 File Dataset for analysis. (SAV)

S6 File Relevant publication. (PDF)
