# Supplementary material for: A multimodal intervention to improve hand hygiene compliance via social cognitive influences among kindergarten teachers in China
Source: PLoS One. 2019 May 14;14(5):e0215824. doi: 10.1371/journal.pone.0215824 (PMC6516664; doi:10.1371/journal.pone.0215824)
Supplement: S1 File — (DOCX) [file pone.0215824.s001.docx]

Protocol for a cluster randomized controlled trial of an intervention to improve hand hygiene and decrease Hand, Foot and Mouth Disease in kindergartens – the “Clean Hands, Happy Life” study.

**Summary**

**BACKGROUND:** Hand, foot and mouth disease (HFMD) has become an epidemic problem within Mainland China over the last decades, with annual outbreaks across the country. Care focuses on symptom relief however complications are relatively common and can even be fatal. Children in kindergarten settings are at high risk of contracting the disease where it can spread rapidly, and are especially susceptible to developing complications. Hand hygiene (HH) is among the simplest and most effective preventive measures to reduce these infections. However, compliance with hand hygiene among children and teachers attending kindergartens is consistently suboptimal in China. We developed a culturally-tailored intervention based on a number of successful Dutch school-based hand hygiene interventions, and aim to pilot the intervention in kindergartens of Shenzhen to improve the HH compliance of kindergarten children and teachers, and eventually decrease HFMD and relevant complications in China.

**METHODS:** A cluster randomized controlled trial with kindergartens as the unit of randomization will be conducted from May till October in 2015. Intervention kindergartens will receive: i) HH products with refills for six months: soap dispensers, towel dispensers, paper towels and soap ii) Reminders and cues for action, including posters, stickers, HH-related reading books, memory games, coloring pages and diploma designated for children iii) one kick-off event with four stations, including hand hygiene e-game with tablets, hand washing instructions, educational story time, and diploma awards iv) education training for kindergarten teachers and parents about the national guideline of HH in kindergartens. Two intervention arms will be assigned in this study: i) kindergartens only and ii) kindergarten and family, with which additional health education materials contribution and training for children’s parents and/or guardians, while one control arm continues usual practice during the six months but receive the intervention package after the study.

We calculated the sample size taking into account the intracluster correlation coefficient, the cost per cluster and cost per person, the expected effect in behavior change, the power and the significance level of the study. We assumed an ICC=0.1, and specified the cost per cluster 2500 RMB (360 US dollar) and the cost per person 10 RMB (1.5 US dollar), the power 90% and a significance level of 0.05(two-side). This resulted a minimum of 18 clusters and 48 children for each cluster to detect the expected compliance with HH guideline increased from 30% (baseline data, unpublished) to 80%. We will use simple random allocation to select kindergartens to represent three types of kindergarten in urban or semi-urban areas for each of the intervention and control arms.

The primary outcome is HFMD incidence rate and absence due to sickness, which will be measured using a self-designed absence card and monitored throughout the intervention by parents and kindergarten doctors. The second outcome is HH compliance of children and teachers measured using ODK-based observation data collector. Observation will be taken place at baseline, 1 month, 3 months and 6 months follow up. In addition, teachers socio-cognitive factors of HH behaviors in intervention arm will be measured using a self-designed questionnaire at baseline and 6 months follow up. Process evaluations will be embedded within the trial.

Data will be analyzed using SPSS version 20 (SPSS Inc., USA) and R version 2·12·2 (R Foundation, Austria). One-way analysis of variance with Chi-Square and analysis of variance will be used to compare baseline characteristics after clustering the data by kindergartens. The incidence rate of diseases will be calculated during six-month follow-up in two intervention arms and the control arm. Episodes of illness which start on the first day that parents and/or school doctors start monitoring disease incidence will be excluded. Incidence rate ratios and compliance rate with 95% confidence intervals will be obtained to compare the effect of two intervention arms with the control arm. Additional linear regression analyses will be performed to compare the effect of the intervention arms on the absence duration and compliance rate for all cases corrected by the area and type of the kindergartens, HH specific moments, physical environment and follow-up duration.

**DISCUSSION:** This project will establish the effectiveness of an intervention that supports kindergarten infection prevention, HFMD in particular, decrease children’s absence due to sickness, and improves HH behaviors in China. It will also provide information regarding intervention development and implementation.

**TRIAL REGISTRATION:** The Ethics Committee of Shenzhen Center for Disease Control and Prevention Trial Number: 2015005, and Netherlands Trial Registered TC – 5395.

**深圳市幼儿园手部卫生促进项目实施方案**

**“洁净双手，快乐生活”**

深圳市疾病预防控制中心

深圳市南山区疾病预防控制中心

深圳市宝安区疾病预防控制中心

Erasmus University Medical Center Rotterdam

2015年3月25日

**目 录**

[**一、项目背景** 1](#_Toc414350784)

[**二、技术路线** 3](#_Toc414350785)

[**三、项目启动** 6](#_Toc414350786)

[（一）前期准备 6](#_Toc414350787)

[（二）正式启动 6](#_Toc414350788)

[（三）预实验 7](#_Toc414350789)

[**四、基线调查** 9](#_Toc414350790)

[（一）幼儿园教师手部卫生行为依从性观察 9](#_Toc414350791)

[（二）幼儿园教师手部卫生情况问卷调查 9](#_Toc414350792)

[（三）手部卫生相关环境因素调查 9](#_Toc414350793)

[（四）幼儿园儿童基本情况问卷调查 9](#_Toc414350794)

[（五）幼儿园儿童传染病发病率监测 10](#_Toc414350795)

[**五、干预实施** 9](#_Toc414350796)

[（一）发放洗手用品 9](#_Toc414350797)

[（二）发放健康教育材料 9](#_Toc414350798)

[（三）环境表面清洁 9](#_Toc414350799)

[（四）健康教育课程 10](#_Toc414350800)

[（五）团队培训课程 10](#_Toc414350801)

[**六、效果评价** 9](#_Toc414350802)

[（一）幼儿园教师手部卫生行为依从性 9](#_Toc414350803)

[（二）幼儿园儿童传染病发病率 9](#_Toc414350804)

[**七、经费预算** 10](#_Toc414350805)

[**附件** 11](#_Toc414350806)

[附件1 深圳CDC与Erasmus MC手部卫生促进项目合作协议 11](#_Toc414350807)

[附件2 荷兰专家在深圳一周日程安排表 12](#_Toc414350808)

[附件3 深圳市幼儿园手部卫生依从性观察表（手机APP文本） 14](#_Toc414350809)

[附件4 深圳市幼儿园教师手部卫生情况调查问卷 17](#_Toc414350810)

[附件5 深圳市幼儿园儿童基本情况调查表 22](#_Toc414350811)

[附件6 幼儿园儿童缺勤调查表 23](#_Toc414350812)

[附件7 瑞典SCA公司洗手用品供应方案 24](#_Toc414350813)

[附件8 干预中使用的健康教育材料一览 25](#_Toc414350814)

# 一、项目背景

手足口病是儿童常见传染病之一，近年来深圳市手足口发病率逐年增高，给儿童、家庭和社会造成了沉重的疾病负担。当前对手足口病缺乏疫苗和特异性防制手段，是造成手足口病发病率居高不下的主要原因。多项研究表明手部卫生习惯对手足口病的发生有显著影响，但国内尚未见有对儿童进行手部卫生干预并能科学评价干预效果的研究报道。幼儿园儿童为手足口病的高发人群，幼儿园教师的手部卫生行为一方面会影响到幼儿园儿童的手部卫生行为，另一方面也可能造成细菌和病毒的传播。故幼儿园是防治手足口病的重要场所。

荷兰伊拉斯姆斯大学于2009年~2013年进行一项旨在改善幼儿园教师手部卫生行为依从性、降低幼儿园儿童传染病发病率的研究。该研究是全球首个观察幼儿园教师及儿童手部卫生行为依从性、使用儿童胃肠道及呼吸道感染的发病率来评价手部卫生干预效果的研究。该研究不同于传统的健康教育研究，其先进性在于使用依从率和发病率来定量评价研究结果，并且使用社会认知理论及阶梯式行为改变方法来量体裁衣的制定针对幼儿园教师的手部卫生促进策略。

该研究耗资50万欧元的，持续5年，分为两个阶段：第一阶段是调查幼儿园教师手部卫生行为的现状及影响因素，共观察到350名幼儿园教师的2001次手部卫生行为，其中符合规范的仅有841次（手部卫生依从率为42%）。第二阶段是在荷兰的四个城市选取71家幼儿园，进行随机对照试验干预。干预组（36家）接受包含四种要素的干预：发放洗手用品；手部卫生知识培训；团队培训；发放手部卫生健康教育材料，而对照组（35家）不施加任何干预措施。通过观察教师及儿童的手部卫生行为依从性及监测儿童在干预过程中的胃肠道和呼吸道传染病发病率来评价干预效果。研究结果表明环境因素和社会认知因素均会影响教师和儿童的手部卫生行为，针对这些影响因素制定干预策略，在幼儿园开展手部卫生促进项目，可显著改进幼儿园教师的手部卫生行为、降低幼儿园儿童的传染病发病率。

深圳市疾病预防控制中心于2012年与荷兰伊拉斯姆斯大学（Erasmus University）公共卫生学院建立起长期合作关系，于2015年共同在深圳市幼儿园开展手部卫生促进项目。项目采取荷兰伊拉斯姆斯大学专家提供的先进技术，并由瑞典爱生雅公司（SCA）及其子公司中国维达（Vinda）为参与项目幼儿园的所有班级及部分家庭免费提供半年洗手用品。项目在南山区和宝安区抽取18家幼儿园并随机分为3组：幼儿园干预组、幼儿园及家庭干预组和对照组。幼儿园干预组仅在幼儿园内，对教师和儿童进行干预；幼儿园及家庭干预组还在家庭中、对家长进行干预。开展干预前先进行基线调查，基线调查主要包括对幼儿园教师的手部卫生行为观察、对幼儿园儿童传染病发病率监测和对手部卫生相关环境因素的评估三方面内容。完成基线调查后立即开展干预，干预策略包含5项要素：（1）发放洗手用品，并在半年的干预期内维持供应；（2）发放健康教育材料；（3）表面清洁；（4）对幼儿园教师提供一次健康教育课程；（5）对幼儿园教师提供两次团队培训课程。在进行干预的半年时间内持续监测干预组和对照组的两项指标，进行同期比较以评估干预效果：（1）幼儿园教师手部卫生行为依从性；(2)幼儿园儿童传染病发病率。

项目通过幼儿园中开展手部卫生促进项目，发展一种简便易行的干预措施以有效降低幼儿园儿童传染病发病率，预期可在在深圳市所有幼儿园中推广。同时项目制定的各种健康教育材料及手部卫生促进策略也能用于手足口病高发社区的散居儿童，切实降低深圳市儿童的手足口病发病率。项目取得的成果和经验可为全国手足口病防制工作提供借鉴和参考。

# 二、技术路线

项目技术路线如图1所示，项目实施进度表见表1，项目组成员名单见表2。

图1 项目技术路线图

表1 项目实施进度表

| 日期 | 主题 | 内容 |
| --- | --- | --- |
| 3月30日 | 荷兰及SCA专家到达深圳 | 负责培训调查员及健康教育课程讲师 |
| 3月31日 | 项目启动会 | 介绍项目，合影，挂牌 |
| 4月1日  4月~3日 | 预实验 | 培训调查员 |
|  |  | 健康教育培训及示范 |
|  |  | 试填调查问卷 |
| 4月7日~  5月4日 | 基线调查  一次手部卫生观察 | 幼儿园教师手部卫生行为依从性观察（1） |
|  |  | 幼儿园教师手部卫生情况问卷调查 |
|  |  | 手部卫生相关环境因素调查 |
|  |  | 幼儿园儿童基本情况问卷调查 |
|  |  | 幼儿园儿童传染病发病率监测 |
| 4月16日~  5月4日 | 开展干预  （1~2天内在一家幼儿园开展4项干预策略） | 发放洗手用品 |
|  |  | 发放健康教育材料 |
|  |  | 开始环境表面清洁，持续至干预结束 |
|  |  | 给幼儿园教师提供1小时健康教育课程 |
| 5月4日~  5月27日 | 第二次手部卫生观察 | 幼儿园教师手部卫生行为依从性观察（2） |
| 5月13日~  5月28日 | 团队培训 | 给幼儿园教师提供1小时团队培训 |
| 5月29日~  6月24日 | 第三次手部卫生观察 | 幼儿园教师手部卫生行为依从性观察（3） |
| 9月2日~  9月9日 | 小规模干预 | 简单健康教育课程并调查儿童在暑假期间是否有发病 |
| 10月1日 | 结束干预 | 停止补充洗手材料 |
| 10月4日~  10月25日 | 第四次手部卫生观察 | 幼儿园教师手部卫生行为依从性观察（4） |
| 12月31日 | 结束发病率监测 |  |

表2 项目组成员名单

| 单位 | 领导小组 | 项目组成员 |
| --- | --- | --- |
| 深圳市卫生和计划生育委员会 | 张丹，市卫计委副主任；  张英姬，疾控处处长 |  |
| 深圳市疾病预防控制中心 | 程锦泉，中心主任 | 赵志广，谢旭，侯万里，周彦，  张振 |
| 南山区疾病预防控制中心 | 孙健，中心主任 | 戴传文，黄燕飞，翟禹涵 |
| 宝安区疾病预防控制中心 | 周指明，中心主任 | 马智超，熊田甜 |
| 荷兰伊拉斯姆斯大学 | Prof. Dr. J.H. Richardus，  公共卫生学院教授 | Vicki，Nana， Elise， Kylah |
| 瑞典爱生雅公司 | Peter Blomstrom，  全球新兴业务总监 | Hallie Huang |
| 调查员 | 蔡亚丽，董方圆，符霞，李山，林莹，杨品超，黄燕飞，  翟禹涵 | |

# 三、项目启动

## （一）前期准备

采取分层整群随机抽样技术，在南山区和宝安区抽取18家幼儿园开展手部卫生促进随机对照试验，选取幼儿园全部班级的儿童和教师均纳入干预，合计约7000余名幼儿园儿童。其中实验组分为“幼儿园干预组”（6家）和“幼儿园及家庭干预组”（6家），并设置对照组（6家）。幼儿园干预组为仅在儿童在校时间内在幼儿园开展手部卫生促进活动，幼儿园及家庭干预组为在幼儿园和家庭同时开展手部卫生促进活动，对照组不采取任何干预措施。

表3 分层整群随机抽样表

| 地区 | 服务人群 | 幼儿园干预组 | 幼儿园与家庭干预组 | 对照组 |
| --- | --- | --- | --- | --- |
| 南山区 | 机关事业单位 | 蓓蕾幼儿园  省级，公办  390人 | 西丽幼儿园  省级，公办  480人 | 南山区机关幼儿园  省级，公办  700人 |
|  | 较高收入人群 | 深圳大学幼儿园  市级，民办  300人 | 蔚蓝海岸幼儿园  省级，民办  320人 | 育才三幼  市级，民办  360人 |
|  | 流动人口 | 宝乐幼儿园  无级别，民办  300人 | 海滨幼儿园  区级，民办  300人 | 沙河中心幼儿园  区级，民办  350人 |
| 宝安区 | 机关事业单位 | 宝安区机关幼儿园  省级，公办  640人 | 建安新村幼儿园  省级，公办  460人 | 兴华幼儿园  省级，公办  515人 |
|  | 较高收入人群 | 特蕾新幼儿园  市级，民办  380人 | 海丽达幼儿园  省级，民办  300人 | 凯旋城幼儿园  市级，民办  390人 |
|  | 流动人口 | 小博士幼儿园  区级，民办  460人 | 翻身幼儿园  区级，民办  330人 | 福慧幼儿园  民办，无级别  250人 |

## （二）正式启动

定于2015年3月31日上午召开项目启动会，参会人员包括：市卫计委领导；市CDC领导及项目组成员；南山区、宝安区CDC领导及项目组成员；南山区、宝安区教育局领导；荷兰伊拉斯姆斯大学专家4名；瑞典SCA公司专家2名；维达公司（SCA子公司）专家2名；18家幼儿园园长及校医。SCA及维达公司还邀请了7家媒体进行采访。

启动会内容主要是对项目内容进行简要介绍，对18家幼儿园授牌“深圳疾病预防控制中心-荷兰Erasmus大学 健康促进项目示范点”并合影，及接受媒体采访。启动会时对校医发放《幼儿园儿童发病或缺勤情况监测报告卡》，并说明填报的注意事项。启动会结束后根据相应幼儿园人数向校医发放《深圳市幼儿园儿童基本情况调查表》，要求由各班教师发放给家长填写后，由校医收集回传至市CDC。

## （三）预实验

在正式干预纳入的18家幼儿园之外再选取一家幼儿园，开展3天预实验，主要包含如下内容：

**1. 培训调查员**

在市CDC的硕士研究生中选取7人作为调查员，并由南山区、宝安区各派出2名工作人员作为调查员，参与对本区幼儿园的调查，观察幼儿园教师手部卫生行为依从性。由荷兰专家对11名调查员在预实验现场进行培训，直到其对同一对象的观察结果一致性>70%；

**2. 健康教育课程培训**

干预需对幼儿园教师进行一次健康教育课程及一次小组培训，每次培训均耗时一小时，培训讲师由市CDC公共卫生医师担任，并且在预实验中由荷兰专家指导，试讲课程。

预实验具体日程安排**见附件2**。

# 四、基线调查

## （一）幼儿园教师手部卫生行为依从性观察

由接受过统一培训的9名调查员观察幼儿园教师的手部卫生行为。每家幼儿园观察9名教师，每名教师由1名观察员连续观察2小时，观察其在各种场所中与手部卫生行为相关的活动，包括在教室、厨房、午休场所及操场等处。调查员记录手部卫生行为发生的次数以及其中符合规范行为的次数，计算手部卫生行为依从性率。

为保持观察的隐蔽性，到达幼儿园后，观察员将告知教师观察的是总体卫生情况，而非教师的手部卫生行为。同时使用手机记录观察数据，目前已与荷兰Erasmus大学合作，开发了用于安卓系统手机的中文App应用程序，可记录观察数据并将其汇总，已顺利调试及测试。手部卫生行为观察表的文本**见附件3**。

## （二）幼儿园教师手部卫生情况问卷调查

调查员观察完每名幼儿园教师的手部卫生行为之后，要求其填写《深圳市幼儿园教师手部卫生情况调查问卷》（**见附件4**），调查问卷内容包括个人基本情况，对手部卫生的知识、信念、行为等。问卷可由幼儿园教师自行填写，由调查员负责质量控制，发现漏项或是不合格之处要求其补填。

## （三）手部卫生相关环境因素调查

调查员观察完每名幼儿园教师的手部卫生行为之后，也对其所在教室的手部卫生相关环境因素进行评估和记录，主要包括如下数据：教师数，儿童数，儿童年龄范围，水龙头数，毛巾数量及类型，肥皂数量及类型，酒精洗手液数量及类型，洗手液盒是否及时填充，等。环境因素调查同样使用手机应用程序记录，调查表文本**见附件3**。

## （四）幼儿园儿童基本情况问卷调查

考虑不同家庭背景情况、不同性别、不同年龄的幼儿园儿童，其手部卫生习惯及各种相关传染病的发病率存在差异，故需要对纳入干预幼儿园儿童的基本情况进行调查，建立基线资料数据库。以班级为单位，由各班班主任向家长发放《深圳市幼儿园儿童基本情况调查表》（**见附件5**），家长填写后由校医负责收回、核查、回传至市CDC。

## （五）幼儿园儿童传染病发病率监测

干预是否能有效减少幼儿园儿童传染病发病率，是评价干预效果的重要指标。研究在采取干预措施前即开始对幼儿园儿童的传染病发病率进行监测，直至干预结束后方结束监测，以幼儿园儿童传染病发病率的变化情况做评价干预效果的主要指标。

**（1）具体做法：**由班主任记录各自班级每天体检异常儿童或缺勤儿童的相关信息，填写《幼儿园儿童发病或缺勤情况调查表》（**见附件6**），并在儿童发病三天后随访家长，了解就诊过程及诊断结果，补填相关信息。由校医负责收集及审核报告卡，每两周一次将报告卡回传至市CDC。同时挑选部分配合度较好的班级作为试点，由教师每周拍摄一次全班所有儿童的手部照片，回传至市CDC。项目结束时进行回顾性分析，作为判断手足口病的依据之一。

**（2）监测报告卡：**主要包括两方面的内容：一是儿童发病当天出现的症状和体征，由教师当天填写，二是儿童的诊断结果，由教师三天后随访家长填写。故监测的传染病不仅限于手足口病，也包括感冒、腹泻、麻疹等儿童常见传染病。

**（3）质量控制措施：**按有效报告例数向教师及校医发放奖励（有效报告指即使是未明确诊断的疾病或是儿童因家中有事请假缺勤，只要校医真实、准确的填写监测报告卡，均算作有效）；按月收集幼儿园儿童缺勤记录表，与校医报告情况核对，查漏补缺；按月对校医上报的监测报告卡随机抽查，直接联系家长核实相关信息，确保记录真实可信。

# 五、干预实施

干预策略主要包含发放洗手用品、一次知识培训、两次小组培训、发放健康教育材料和表面消毒五部分。其中知识培训和小组培训在干预组的幼儿园教师开展，

通过随机分层整群抽样方法将18家幼儿园随机分为3组：幼儿园干预组（6家），幼儿园及家庭干预组（6家）和空白对照组（6家）。两组干预组的区别在于，“幼儿园干预组”仅在幼儿园内发放洗手用品和健康教育材料，而“幼儿园+家庭干预组”还向家长发放洗手用品和健康教育材料，让家长带回家中使用。干预措施包含如下5项具体策略：

## （一）发放洗手用品

在幼儿园放置纸巾、洗手液、肥皂、酒精净手装置等洗手用品，并及时补充。对家庭干预组，以班级为单位，向家长发放洗手用品及健康教育材料，供其在家中使用。以上洗手用品均由瑞典SCA公司和维达公司免费提供。产品样图**见附件7**。

发放洗手用品在每家幼儿园完成基线调查之后立即开展。按照12家干预组幼儿园每间教室配备2个擦手纸分配器、2个泡沫洗手液分配器，并由SCA负责保证上述幼儿园洗手液和擦手纸从2015年4月~10月得到足量供应。6家对照组幼儿园在干预期间并不供应洗手用品，但是会在干预后按照同样标准补偿半年洗手用品供应。

## （二）发放健康教育材料

制作面向幼儿园教师和幼儿园儿童的手部卫生健康教育材料，在幼儿园内张贴洗手知识海报和提示，给校医及幼儿园教师发放洗手及防病知识的培训手册。在家庭干预组中还加入以下措施：给家长发放手部卫生知识宣传手册，并发放可供家长与儿童一起阅读的手部卫生知识小故事、连环画等。干预中使用的各种健康教育材料**见附件8**。

发放健康教育材料在每家幼儿园完成基线调查之后立即开展。

## （三）环境表面清洁

由幼儿园教师每天使用消毒液擦拭餐桌、床围栏、门把手、水龙头、玩具、厕所表面，进行表面清洁。环境表面清洁在每家幼儿园完成基线调查之后立即开展，一直持续到2015年10月干预结束。

## （四）健康教育课程

对幼儿园教师提供1小时的健康教育课程，主要包括如下内容：传染病的传播途径，手部卫生行为的重要性，何时应洗手及如何洗手。使用PowerPoint幻灯片进行演示讲解，并加入互动环节：使用紫外线呈色手霜（UV Glow Cream）演示洗手的效果（过程见http://www.hand-hygiene.com/uv-germ-range.htm），另外SCA的专家也会带来一些最新的健康教育技术进行示范。在健康教育课程结束后，给幼儿园教师发放手部卫生知识手册，以便他们在课程结束后也能继续学习手部卫生知识。

健康教育课程在每家幼儿园完成基线调查之后立即开展，由市CDC公共卫生医师主讲，每个班级抽取一名幼儿园教师进行培训。

## （五）团队培训课程

对幼儿园教师提供2次团队培训课程，以进一步改进手部卫生。每次培训课程耗时1小时，主要内容如下：由6~10名幼儿园教师组成团队，团队的队长给出任务，这些任务涉及手部卫生的目标、障碍等。培训的最终目标是每个队给出任务的实际解决方案。在第二次团队培训时进行评分，组员们讨论他们团队得到的分数，希望达到的分数，以及怎样才能达到理想的分数。

2次团队培训课程分别在2015年5月和9月开展，由市CDC公共卫生医师主持。从每个班级随机抽取1名教师进行培训。

# 六、效果评价

干预从2015年4月起，至2015年10月结束，持续半年。干预结束后，比较试验组及对照组的如下指标变化情况，评价干预效果：

## （一）幼儿园教师手部卫生行为依从性

由调查员在2015年4月（开展干预前）、5月（开展干预1个月后）、6月（第1次团队培训后）、10月（干预结束后）对幼儿园教师的手部卫生行为进行4次观察，比较依从性变，化以评价干预效果。观察时应用盲法，即幼儿园教师并不知晓研究目的，而被告知调查员观察的是总体卫生情况。

18家幼儿园，每家幼儿园抽取大、中、小3个班级各一，每个班观察3名教师，一家幼儿园观察9名教师，共对幼儿园教师648人次进行观察，每名教师连续观察2小时。

## （二）幼儿园儿童传染病发病率

幼儿园儿童传染病发病率监测从2015年4月起（开展干预前），持续至2015年12月结束。由各班班主任记录每天体检异常儿童或缺勤儿童的相关信息，填写《幼儿园儿童发病或缺勤情况监测报告卡》，监测的传染病不仅限于手足口病，也包括感冒、腹泻、麻疹等儿童常见传染病。由校医收集、核查监测报告卡并定期回传至市CDC。按有效报告例数向教师和校医发放奖励。

同时挑选部分配合度较好的班级作为试点，由教师每周拍摄一次全班所有儿童的手部照片，回传至市CDC。项目结束时进行回顾性分析，作为判断手足口病的依据之一。

# 七、经费预算

| 科目 | 申请经费 | 备注（计算依据和说明） |
| --- | --- | --- |
| 一、研究经费 |  |  |
| **1.科研业务费** | **133000** |  |
| （1）测试/计算 | 50000 | 现场调研费用，资料录入及分析费用 |
| （2）能源/动力费 | 10000 | 交通费用 |
| （3）会议费/差旅费 | 10000 | 召开项目启动会议及18家幼儿园挂牌制作 |
| （4）出版物/文献/信息传播费 | 53000 | 健康教育材料印刷费用按7000名儿童及相应的教师、家长计算，估计为50000元，研究报告制作及印刷费用3000元 |
| （5）其他 | 10000 | 办公用品等 |
| **2.实验材料费** |  |  |
| （1）原材料/试剂/药品购置费 |  | 洗手用品均由爱生雅公司及维达公司免费提供 |
| **3.仪器设备费** | **10000** |  |
| （1）购置 | 10000 | 印刷调查问卷及监测表，购置必要的健康教育器材、打印耗材、设备维修等 |
| （2）试制 |  |  |
| 4.实验室改装费 |  |  |
| **5.协作费** | **136000** | 18家幼儿园，在半年的干预时间内，给校医按照传染病报告的有效例数发放奖励，估计额度约为每人5000元，进行培训的器材、场地费用10000元，给18家幼儿园园长发放协作费每人2000元。 |
| **二、国际合作与交流费** |  |  |
| **三、劳务费** | **32400** | 9名调查员，观察18家幼儿园4次，共观察教师18*9*4=648人次，观察每人次耗时2小时，按每人次50元/人标准发放劳务费，合计为32400元 |
| **四、管理费** |  |  |
| **合计** | **311400** |  |

# 附件

**附件1 深圳CDC与Erasmus MC手部卫生促进项目合作协议**


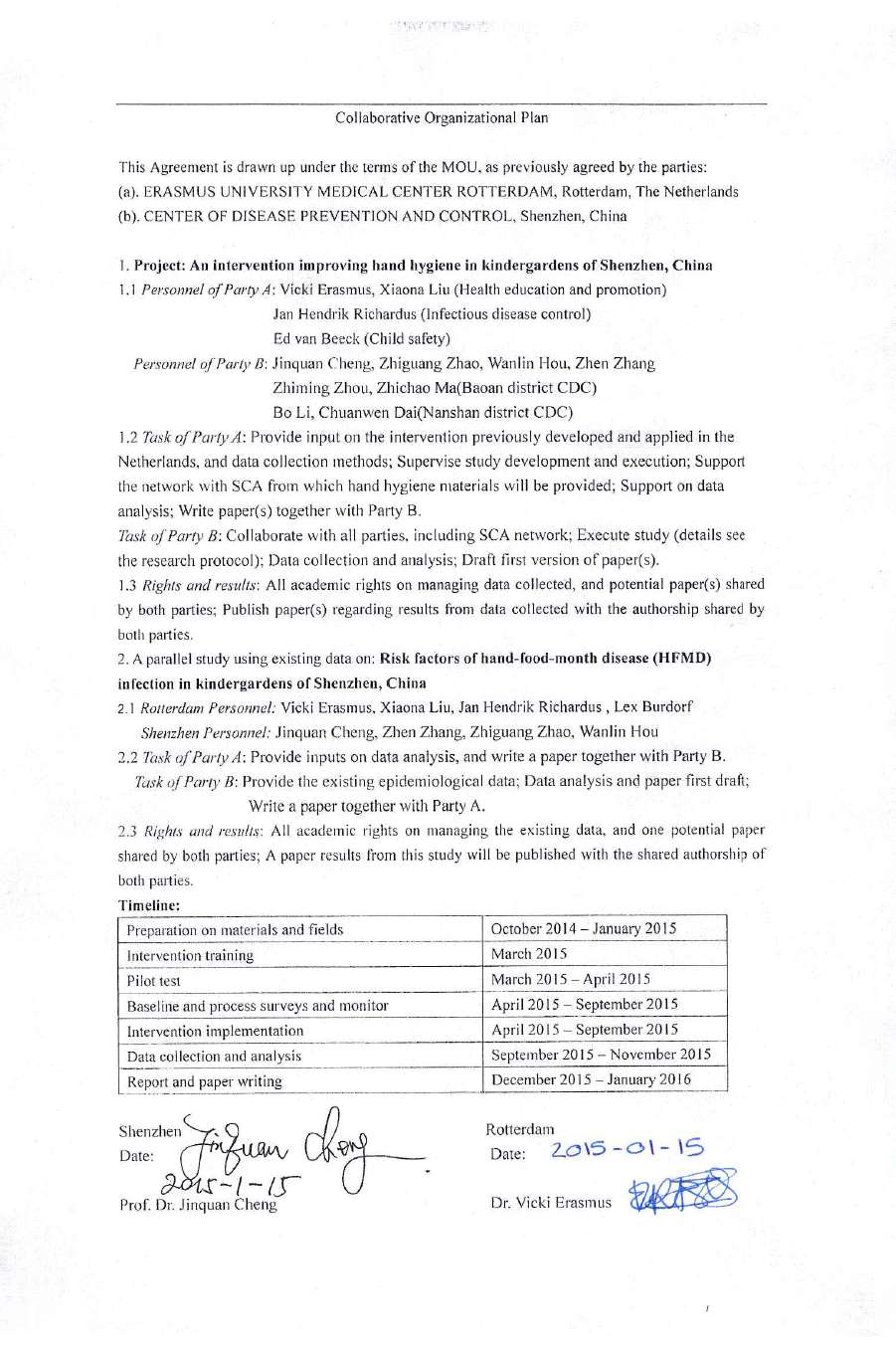


**附件2 荷兰专家在深圳一周日程安排表**

**（2015年3月30日~4月3日）**

| **日期** | **时间** | **活动** | **人员** |
| --- | --- | --- | --- |
| 3月30日 | 上午 | Erasmus大学专家及SCA专家到达香港机场  —安排接机至市CDC | Erasmus大学专家：Vicki，Nana，Elise，Kylah  SCA公司专家：Peter, Hallie |
|  | 下午 | 项目组成员见面会，预实验准备：  —确定活动的日程安排和人员分工  —将各种打印材料整理成包裹以便发放 | 市CDC人员  Erasmus大学专家  SCA专家 |
| 3月31日 | 上午 | 项目启动会  —项目简介（15分钟）  —领导及专家发言（50分钟）  —18家幼儿园授牌及合影（20分钟）  —媒体采访（30分钟）  —培训校医填写监测表（10分钟） | 卫计委领导、市CDC领导及人员  Erasmus大学专家；SCA公司、维达公司专家  南山区、宝安区CDC领导及人员  南山区、宝安区教育局领导  18家幼儿园园长及校医  9家媒体 |
|  | 下午 | 选取一家幼儿园开展预实验，对老师和儿童的知识传播（口头讲解及发放健康教育材料），包括如下活动：  —使用手机演示Ella洗手学园小游戏—和小朋友一起读洗手画册  —示范如何正确洗手  给参加活动的儿童发放洗手学园毕业证 | 市CDC人员  Erasmus大学专家  SCA专家  11名调查员 |
| 4月1日 | 上午 | 前一天预实验的小结会  培训11名调查员，使其熟悉调查要求、调查工具及观察过程 | 市CDC人员  Erasmus大学专家  SCA专家  11名调查员 |
|  | 下午 | 对幼儿园教师的健康教育课程示范，团队培训课程示范  前往预实验幼儿园：  —在教室里对11名调查员进行观察教师手部卫生行为的现场培训，讲解观察中的要点和难点  —SCA专家离开深圳 | 市CDC人员  Erasmus大学专家  SCA专家  11名调查员 |
| 4月2日 | 上午 | 在深圳CDC召集11名调查员开小结会，继续对调查员的培训  —讨论昨天下午现场观察中遇到的问题  —再次进行强化培训 | 市CDC人员  Erasmus大学专家  11名调查员 |
|  | 下午 | 在预实验幼儿园对调查员进行现场培训  —在教室内进行模拟观察  —把模拟观察的过程录像，并回到CDC播放，进行回顾分析，以确定调查员是否培训合格 | 市CDC人员  Erasmus大学专家  11名调查员 |
| 4月3日 | 上午 | 总结会 | 市CDC人员  Erasmus大学专家  11名调查员 |
|  | 下午 | Vicki和Elise离开深圳前往香港  Nana和Kylah将留在深圳协助调查及干预实施直到6月 | 市CDC人员  Erasmus大学专家  11名调查员 |

## 附件3 深圳市幼儿园手部卫生依从性观察表（手机APP文本）

**基本特征**

1. 观察哪个幼儿园？（ ）

2. 观察时间？ 上午 下午

3. 当时有几位教师在场？ （ ）人

4. 当时教室里有多少儿童？（如果确切的数目是未知的，则估计到最近的5个）

5. 在场人的平均年龄是？ 3岁 4岁 5岁

**教室环境评估**

1. 教室表面整体为？ 清洁 脏

2. 当时肥皂设施总数为？

儿童使用的肥皂分配器------儿童使用数量（ ）

教师使用的肥皂分配器------教师使用数量（ ）

共享使用的肥皂分配器------共享数量 （ ）

3. 当时水龙头的总数为？

儿童使用的水龙头------儿童使用数量 （ ）

教师使用的水龙头------教师使用数量 （ ）

共同使用的水龙头------共享数量 （ ）

4. 当时纸巾设施的总数为？

儿童使用的纸巾分配器------儿童使用数量（ ）

教师使用的纸巾分配器------教师使用数量（ ）

共同的纸巾分配器--------共享数量 （ ）

5. 当时教室里有怎样的毛巾？

给每个儿童和教师分别使用的独立毛巾

给儿童使用的共享毛巾和给教师使用的共享毛巾-----为孩子提供的毛巾数量（ ）

为教师提供的毛巾数量 （ ）

给所有儿童和教师使用的共享的毛巾------毛巾总数量 （ ）

**洗手卫生的情景和依从性**

1. 你在哪种情景下进行观察？

在午餐/零食时间

在课堂中

在艺术和手工艺活动中

在外面玩

在运动/体育课中

在午睡或附近时间里

2. 假如你看到了在教师或者外面发生了洗手卫生，该情景涉及哪些人？

儿童 教师

3. 涉及儿童的洗手卫生情景

3.1有什么情况下进行的洗手卫生？

吃饭前 准备食物前 上完厕所后 手明显脏时 在外面玩后

3.2 此情景里孩子洗了他/她的手了么？

是的，双手都洗得很好

是的，但是没有用肥皂

是的，但是没有擦干净

只是冲了手，没有用肥皂也没有擦干手

无，完全没有洗手

3.3 此情景里老师提醒孩子/孩子们洗手了吗？

是的 不是 不清楚

3.4 是有可能的排泄物接触到活动中的面接触？

是的 不是 不清楚

3.5 对该情景/洗手卫生的评论（ ）

----例如：关于在该情景里发生洗手的可行性，或防止洗手的障碍等

4. 涉及教师的洗手卫生情景

4.1 有什么情况下进行的洗手卫生？

准备食物前 供应食物前 在吃自己的食物前 在协助孩子进食前

在帮助孩子使用厕所后 咳嗽/打喷嚏后 可能和体液和/或伤口接触后

和垃圾箱接触后 和脏布接触后 在指导孩子们在外面玩后

手明显脏时 使用清洁用品后

4.2 此情景里孩子们洗了他/她的手了么？

是的，双手都洗得很好

是的，但是没有用肥皂

是的，但是没有擦干净

只是冲了手，没有用肥皂也没有擦干手

无，完全没有洗手

4.3 是有可能的排泄物接触到活动中的面接触？

是的 不是 不清楚

4.4 当时老师佩戴任何防护设备了吗？

是的 不是

4.5 对该情景/洗手卫生的评论（ ）

----例如：关于在改情景里发生洗手的可行性，或防止洗手的障碍等

## 附件4 深圳市幼儿园教师手部卫生情况调查问卷

尊敬的老师：

您好！为了解幼儿园教师在幼儿园工作时间的洗手情况，我中心特开展此次调查，以制订对策促进幼儿园手部卫生，并预防手部相关传染性疾病。该问卷是匿名填写，所有信息会被严格保密，仅被用于科学研究。且所有问题的答案没有对错之分。请您如实填写！

请注意，洗手指使用肥皂和水洗手。问卷中的部分条目可能让你感觉是询问相同的问题，但请你仍然填写这些条目。

完成此问卷需耗时10~15分钟，感谢您的支持和配合！

深圳市疾病预防控制中心

**问题范例1（将选项涂黑）：**

|  | 肯定不会 ←→ 肯定会 | | | | | | |
| --- | --- | --- | --- | --- | --- | --- | --- |
| 1．你明天会骑自行车上班的可能性为？ | 1 | 2 | 3 | 4 | 5 | **6** | 7 |

**问题范例2（在选项后打勾）**

2. 你明天会骑自行车上班吗？ （1）不会√ （2）会

**一．个人基本情况**

1.1 您的性别: (1)男 (2)女

1.2您的年龄：__________ 岁

1.3您从事幼儿园教育工作有多少年了？__________年

1.4您的教育程度: (1) 初中及以下 (2)高中/中专 (3)大专 (4)本科 (5)研究生及以上

1.5是否有14岁以下儿童和您一起居住？ (1)无 (2)有, _____名

1.6您的手是否干燥呢？ (1)从不干燥 (2)有时干燥 (3)一直干燥

1.7您的手是否有湿疹？ (1)从没有 (2)有时会有 (3)一直有

**二．洗手规范**

2.1以下问题有关在不同情况下，您班级儿童和您自己在工作时的洗手频率。频率幅度从0

（从不洗手）到10（总会洗手）。请您在相应频率代表的数字上打勾。

|  | 从不 ←→ 总是 | | | | | | | | | | |
| --- | --- | --- | --- | --- | --- | --- | --- | --- | --- | --- | --- |
| 如下情况您班上儿童洗手的频率 | | | | | | | | | | | |
| 1. 吃饭前 | 0 | 1 | 2 | 3 | 4 | 5 | 6 | 7 | 8 | 9 | 10 |
| 2. 上厕所后 | 0 | 1 | 2 | 3 | 4 | 5 | 6 | 7 | 8 | 9 | 10 |
| 下列情况您自己洗手的频率 | | | | | | | | | | | |
| 3. 在准备午餐之前 | 0 | 1 | 2 | 3 | 4 | 5 | 6 | 7 | 8 | 9 | 10 |
| 4. 给水果削皮之前 | 0 | 1 | 2 | 3 | 4 | 5 | 6 | 7 | 8 | 9 | 10 |
| 5. 用手捂嘴咳嗽或打喷嚏之后 | 0 | 1 | 2 | 3 | 4 | 5 | 6 | 7 | 8 | 9 | 10 |
| 6. 擦鼻涕之后 | 0 | 1 | 2 | 3 | 4 | 5 | 6 | 7 | 8 | 9 | 10 |
| 7. 换尿布之后 | 0 | 1 | 2 | 3 | 4 | 5 | 6 | 7 | 8 | 9 | 10 |
| 10. 接触体液之后（如唾液，呕吐物，血液，伤口，尿液，鼻涕） | 0 | 1 | 2 | 3 | 4 | 5 | 6 | 7 | 8 | 9 | 10 |
| 11. 户外活动之后 | 0 | 1 | 2 | 3 | 4 | 5 | 6 | 7 | 8 | 9 | 10 |
| 12. 接触脏污纺织品之后（如脏衣服毛巾） | 0 | 1 | 2 | 3 | 4 | 5 | 6 | 7 | 8 | 9 | 10 |
| 13. 上厕所之后 | 0 | 1 | 2 | 3 | 4 | 5 | 6 | 7 | 8 | 9 | 10 |
| 14. 准备奶瓶之前 | 0 | 1 | 2 | 3 | 4 | 5 | 6 | 7 | 8 | 9 | 10 |
| 15. 吃饭之前 | 0 | 1 | 2 | 3 | 4 | 5 | 6 | 7 | 8 | 9 | 10 |
| 16. 给儿童喂食之前 | 0 | 1 | 2 | 3 | 4 | 5 | 6 | 7 | 8 | 9 | 10 |
| 17. 给儿童擦鼻涕之后 | 0 | 1 | 2 | 3 | 4 | 5 | 6 | 7 | 8 | 9 | 10 |
| 18. 给儿童擦屁股之后 | 0 | 1 | 2 | 3 | 4 | 5 | 6 | 7 | 8 | 9 | 10 |

2.2 请标出对以下陈述句的同意程度。可选同意程度从1（完全不同意）到7（完全同意）

|  | 完全不同意 ←→ 完全同意 | | | | | | |
| --- | --- | --- | --- | --- | --- | --- | --- |
| 19. 我非常清楚的知道何时应该洗手以及怎样洗手 | 1 | 2 | 3 | 4 | 5 | 6 | 7 |

2.3请判断如下说法是否正确

| 20. 在为儿童准备午餐前必须洗手 | 错误 | 正确 |
| --- | --- | --- |
| 21. 在更换每一片尿布之后都应当洗手 | 错误 | 正确 |
| 22. 洗手时并不是每次都必须使用肥皂 | 错误 | 正确 |
| 23. 给小孩喂食前应该洗手 | 错误 | 正确 |
| 24. 擦鼻涕后不洗手也没关系 | 错误 | 正确 |

2.4请选择如下情况的可能性

|  | 不可能 ←→ 非常可能 | | | | | | | | | | |
| --- | --- | --- | --- | --- | --- | --- | --- | --- | --- | --- | --- |
| 25. 你工作的幼儿园有小孩得传染性疾病（例如腹泻，普通感冒） | 0 | 1 | 2 | 3 | 4 | 5 | 6 | 7 | 8 | 9 | 10 |
| 26. 因为你不洗手，你班上的一名儿童得了传染病 | 0 | 1 | 2 | 3 | 4 | 5 | 6 | 7 | 8 | 9 | 10 |
| 27. 因为你的同事不洗手，你班上的一名儿童得了传染病 | 0 | 1 | 2 | 3 | 4 | 5 | 6 | 7 | 8 | 9 | 10 |

2.5您认为以下情况的严重性如何：

|  | 不严重 ←→ 非常严重 | | | | | | | | | | |
| --- | --- | --- | --- | --- | --- | --- | --- | --- | --- | --- | --- |
| 28. 一名儿童感染了传染病 | 0 | 1 | 2 | 3 | 4 | 5 | 6 | 7 | 8 | 9 | 10 |
| 29. 您自己感染了传染病 |  |  |  |  |  |  |  |  |  |  |  |

2.6 您是如何洗手的？

|  | 从来不洗 ←→ 每次都洗 | | | | | | | | | | |
| --- | --- | --- | --- | --- | --- | --- | --- | --- | --- | --- | --- |
| 30. 所有需要洗手的情况下我都会洗手 | 0 | 1 | 2 | 3 | 4 | 5 | 6 | 7 | 8 | 9 | 10 |
| 31. 当我很忙时每次也会洗手 | 0 | 1 | 2 | 3 | 4 | 5 | 6 | 7 | 8 | 9 | 10 |
| 32. 即使当发生一些突发事件时，比如接电话，孩子争吵等，每次我仍然会洗手 | 0 | 1 | 2 | 3 | 4 | 5 | 6 | 7 | 8 | 9 | 10 |
| 33.当有传染病发生时，每次我都会洗手 | 0 | 1 | 2 | 3 | 4 | 5 | 6 | 7 | 8 | 9 | 10 |

2.7 您认为您的洗手情况：

|  | 次数更少 ←→ 更加频繁 | | | | | | | | | | |
| --- | --- | --- | --- | --- | --- | --- | --- | --- | --- | --- | --- |
| 34. 与您的同事相比 | 0 | 1 | 2 | 3 | 4 | 5 | 6 | 7 | 8 | 9 | 10 |

**三．您对洗手规范的看法**

3.1 您是如何看待洗手规范的？

1. 完全不重要 < 1 2 3 4 5 6 7 > 非常重要

1. 很舒适 < 1 2 3 4 5 6 7 > 很不舒适
2. 并未给我洁净清新的感觉 < 1 2 3 4 5 6 7 > 给我洁净清新的感觉
3. 很容易做到 < 1 2 3 4 5 6 7 > 很难做到
4. 能导致干燥开裂的手 < 1 2 3 4 5 6 7 > 能避免干燥开裂的手

3.2 请回答如下问题：

|  | 肯定不会 ←→ 肯定会 | | | | | | |
| --- | --- | --- | --- | --- | --- | --- | --- |
| 6. 按照规范洗手会花费您过多的时间吗？ | 1 | 2 | 3 | 4 | 5 | 6 | 7 |
| 7. 工作环境展示有洗手规范会让您觉得更卫生吗？ | 1 | 2 | 3 | 4 | 5 | 6 | 7 |

3.3 您会按照规范洗手是因为:

|  | 完全不同意 ←→ 完全同意 | | | | | | |
| --- | --- | --- | --- | --- | --- | --- | --- |
| 8. 我班上的儿童会减少感染疾病的风险（比如腹泻，普通感冒等） | 1 | 2 | 3 | 4 | 5 | 6 | 7 |
| 9. 我自己感染传染病的风险会变小 | 1 | 2 | 3 | 4 | 5 | 6 | 7 |

3.4 请标出您对以下说法的同意程度:

|  | 完全不同意 ←→ 完全同意 | | | | | | |
| --- | --- | --- | --- | --- | --- | --- | --- |
| 10. 洗手只能减少细菌传播，但并不能减少病毒传播 | 1 | 2 | 3 | 4 | 5 | 6 | 7 |
| 11. 我并不是经常洗手，这也没有太大关系，因为这会增强儿童的抵抗力 | 1 | 2 | 3 | 4 | 5 | 6 | 7 |
| 12. 我不洗手的话会对儿童带来不良影响 | 1 | 2 | 3 | 4 | 5 | 6 | 7 |

**四．洗手行为**

4.1当面临如下情况是，您是否会按照规范洗手？

|  | 肯定不会 ←→ 肯定会 | | | | | | |
| --- | --- | --- | --- | --- | --- | --- | --- |
| 1. 需要洗手的所有时刻 | 1 | 2 | 3 | 4 | 5 | 6 | 7 |
| 2. 工作压力很大的时候 | 1 | 2 | 3 | 4 | 5 | 6 | 7 |
| 3. 当你手部干燥开裂的时候 | 1 | 2 | 3 | 4 | 5 | 6 | 7 |
| 4. 当你的同事都在洗手的时候 | 1 | 2 | 3 | 4 | 5 | 6 | 7 |

4.2在以后

|  | 完全不同意←→ 完全同意 | | | | | | |
| --- | --- | --- | --- | --- | --- | --- | --- |
| 5. 我计划在任何需要洗手的情况下按照规范洗手 | 1 | 2 | 3 | 4 | 5 | 6 | 7 |
| 6. 我将在任何需要洗手的情况下按照规范洗手 | 1 | 2 | 3 | 4 | 5 | 6 | 7 |

4.3按照规范去洗手：

|  | 完全不同意 ←→ 完全同意 | | | | | | |
| --- | --- | --- | --- | --- | --- | --- | --- |
| 7. 是我自然能做到的事 | 1 | 2 | 3 | 4 | 5 | 6 | 7 |
| 8. 是我自觉能做到的事 | 1 | 2 | 3 | 4 | 5 | 6 | 7 |
| 9. 是我需要努力去做到的事 | 1 | 2 | 3 | 4 | 5 | 6 | 7 |
| 10. 是我日常工作和生活的一部分 | 1 | 2 | 3 | 4 | 5 | 6 | 7 |
| 11. 是我不加思考能做到的事 | 1 | 2 | 3 | 4 | 5 | 6 | 7 |
| 12. 是我本来就一直做的事 | 1 | 2 | 3 | 4 | 5 | 6 | 7 |
| 13. 是我会做因为本就应当做的事 | 1 | 2 | 3 | 4 | 5 | 6 | 7 |

4.4你是如何看待鼓励儿童洗手的

|  | 很困难 ←→ 很容易 | | | | | | |
| --- | --- | --- | --- | --- | --- | --- | --- |
| 14. 我认为鼓励儿童洗手 | 1 | 2 | 3 | 4 | 5 | 6 | 7 |
| 15. 当父母并不重视时，我认为鼓励儿童洗手 | 1 | 2 | 3 | 4 | 5 | 6 | 7 |

**五．你所在幼儿园情况**

这部分关于您工作的幼儿园的一系列情况。请标出您对以下说法的同意程度。

|  | 完全不同意 ←→ 完全同意 | | | | | | |
| --- | --- | --- | --- | --- | --- | --- | --- |
| 1. 我们园长认为我必须按照规范经常洗手 | 1 | 2 | 3 | 4 | 5 | 6 | 7 |
| 2. 我的两名同事认为我应当按照规范经常洗手 | 1 | 2 | 3 | 4 | 5 | 6 | 7 |
| 3. 当我同事按照规范洗手时我感到有压力 | 1 | 2 | 3 | 4 | 5 | 6 | 7 |
| 4. 我的同事都按照规范洗手 | 1 | 2 | 3 | 4 | 5 | 6 | 7 |
| 5 我很重视我同事对按照规范洗手的看法 | 1 | 2 | 3 | 4 | 5 | 6 | 7 |
| 6. 如果我的同事不按照规范洗手，我也不洗 | 1 | 2 | 3 | 4 | 5 | 6 | 7 |
| 7. 洗手是每个人的责任 | 1 | 2 | 3 | 4 | 5 | 6 | 7 |
| 8. 我们幼儿园已有洗手所需全部的材料和设备 | 1 | 2 | 3 | 4 | 5 | 6 | 7 |
| 9. 洗手后我用毛巾擦手，而不是用纸巾 | 1 | 2 | 3 | 4 | 5 | 6 | 7 |
| 10. 用酒精洗手器洗手与肥皂和水一样有效 | 1 | 2 | 3 | 4 | 5 | 6 | 7 |

**六．你对本问卷和洗手还有其他意见或建议吗？请写下你的看法：**

**附件5 深圳市幼儿园儿童基本情况调查表**

尊敬的家长：

您好！为了防制手足口病，保护儿童健康，我中心正在开展一项调查，所收集的信息将仅被用作分析儿童手足口病的发生情况，以制订防治对策，不会向第三方泄露，请您如实回答以下问题。感谢支持！ ——深圳市疾病预防控制中心

家长姓名：_____________ 家庭住址：__________________________

家庭座机：_____________ 家长手机：___________ 电子邮件：_____________

儿童姓名：_____________ 儿童性别：___________ 儿童年龄：____岁

1. 是否为单亲家庭 (1)是 (2)否

2. 父亲职业为______；母亲职业为_______（请填写以下数字）

(1)公务员 (2)事业单位职员 (3)企业职员 (4)商业/服务业员工 (5)工人

(6)农民 (7)个体工商户 (8)自由职业 (9)无业或全职在家 (10)其他___________

3. 您的家庭每月平均收入

(1)5000元以下 (2)5000~1万元 (3)1万~3万元 (4)3万元及以上

4. 您目前的户口类型是

(1)深圳户口 (2)无深圳户口，但在深圳居住时间≥6个月

(3)无深圳户口，在深圳居住时间<6个月

5. 您的现居地类型为？

(1)住宅小区 (2)城中村 (3)城乡结合部 (4)其他

以下问题是关于您正在上幼儿园的孩子：

6. 您的孩子是否患慢性疾病（如过敏，哮喘，支气管炎，乳糖不耐受症，甲状腺疾病等）

(1)有，他/她患有_______________ (2)无

7. 您的孩子最近是否在服药 (1)有，他/她服用了_______________ (2)无

8. 他/她是否有兄弟姐妹（仅指在现居地一起生活的）

(1)有，分别是___岁 ___岁 ___岁 (2)无

9. 您的孩子在家主要由谁照看？

(1)父母 (2)祖父母 (3)亲属 (4)保姆 (5)其他

10. 主要照看人的文化程度是？

(1)文盲 (2)小学 (3)初中 (4)高中/中专 (5)本科/大专 (6)研究生及以上

11. 接下来6个月中是否会有搬家、转学或休学一段时间的计划？

(1)有 (2)无

**附件6 幼儿园儿童缺勤调查表**

儿童姓名 ____________ 所在班级 ____________

家长姓名 ____________ 家长联系方式________________

1. 缺勤日期 ___月___日 至 ___月___日

2. 缺勤原因 1. 生病 2. 其他原因，请注明 __________________

***缺勤当天出现的症状和体征（缺勤当天观察儿童或询问家长填写）***

3. 出现症状描述（请观察儿童，或详细询问家长，直接在以下项目中勾选）

(1) 发热 (2) 咳嗽 (3) 鼻塞 (4) 流涕 (5) 咽痛 (6) 头痛 (7) 呕吐 (8) 腹痛

(9) 腹泻 (10) 抽搐 (11)食欲不振 (12)皮肤瘙痒 (13)口唇紫绀

4. 是否出疹：

(1)手 (2)足 (3)口 (4)臀 (5)四肢 (6)躯干 (7)其他部位_________ (8)未出疹

5. 是否有口部疱疹或溃疡

(1) 颊部 (2) 咽峡部 (3) 其他_________ (4) 无

***就医诊断结果（缺勤当日起三天后随访家长，补填以下信息）***

6. 是否就医：

(1) 当天就医 (2) 隔日就医 (3)未就医，原因是____________________________

7. 就医机构1 _____________ 就医日期1 ___月___日 至 ___月___日

诊断结果1 ______________

8. 就医机构2 _____________ 就医日期2 ___月___日 至 ___月___日

诊断结果2 ______________

报告单位 ________________

报告人签名 ________________

报告人联系方式 ________________

报告日期 ________________

**附件7 瑞典SCA公司洗手用品供应方案**


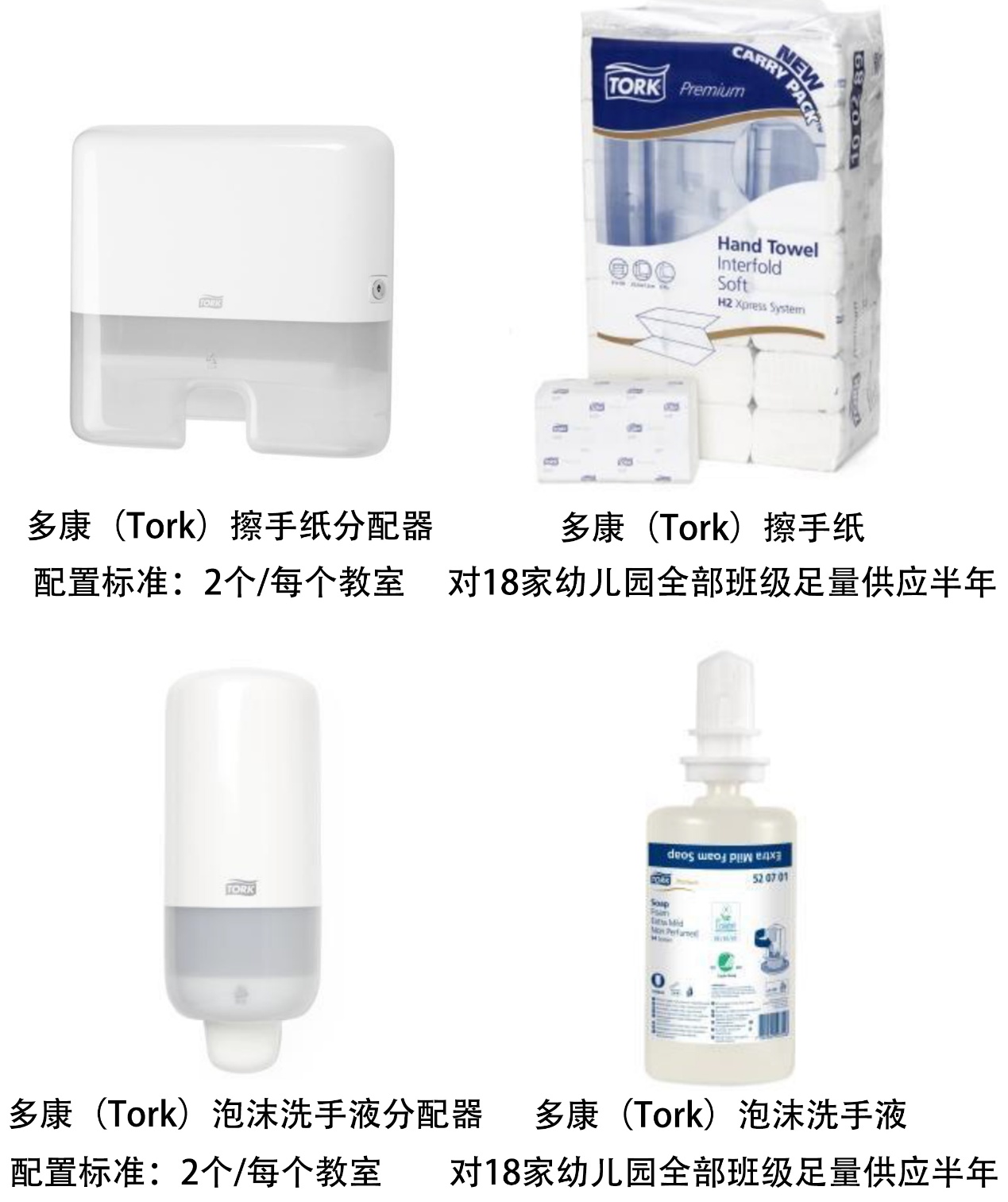


**附件8 干预中使用的健康教育材料一览**

**荷兰Erasmus大学提供的健康教育材料**

1. 海报 - 何时要洗手（儿童版）(张贴于幼儿园和家中)

2. 海报 - 儿童洗手步骤图 (张贴于幼儿园和家中)

3. 海报 – 何时要洗手（成年人版）(张贴于幼儿园和家中)

4. 海报 – 成年人洗手步骤图 (张贴于幼儿园和家中)

5. 手部卫生知识手册 -翻译制作中（给幼儿园老师和家长）

6. 培训使用的PPT –翻译制作中（给幼儿园老师）

**瑞典SCA公司提供的健康教育材料**

7. Ella和小伙伴们洗手奇遇记（画册）（给家长和儿童）

8. Ella洗手学园（手部卫生知识手册）（给家长和儿童）

9. Ella洗手学园毕业证（给儿童）

10. 手部卫生知识折页（给幼儿园老师和家长）

11. Ella应用程序（见网站）（给家长和儿童）

**1. 海报 - 何时要洗手（儿童版）**

**
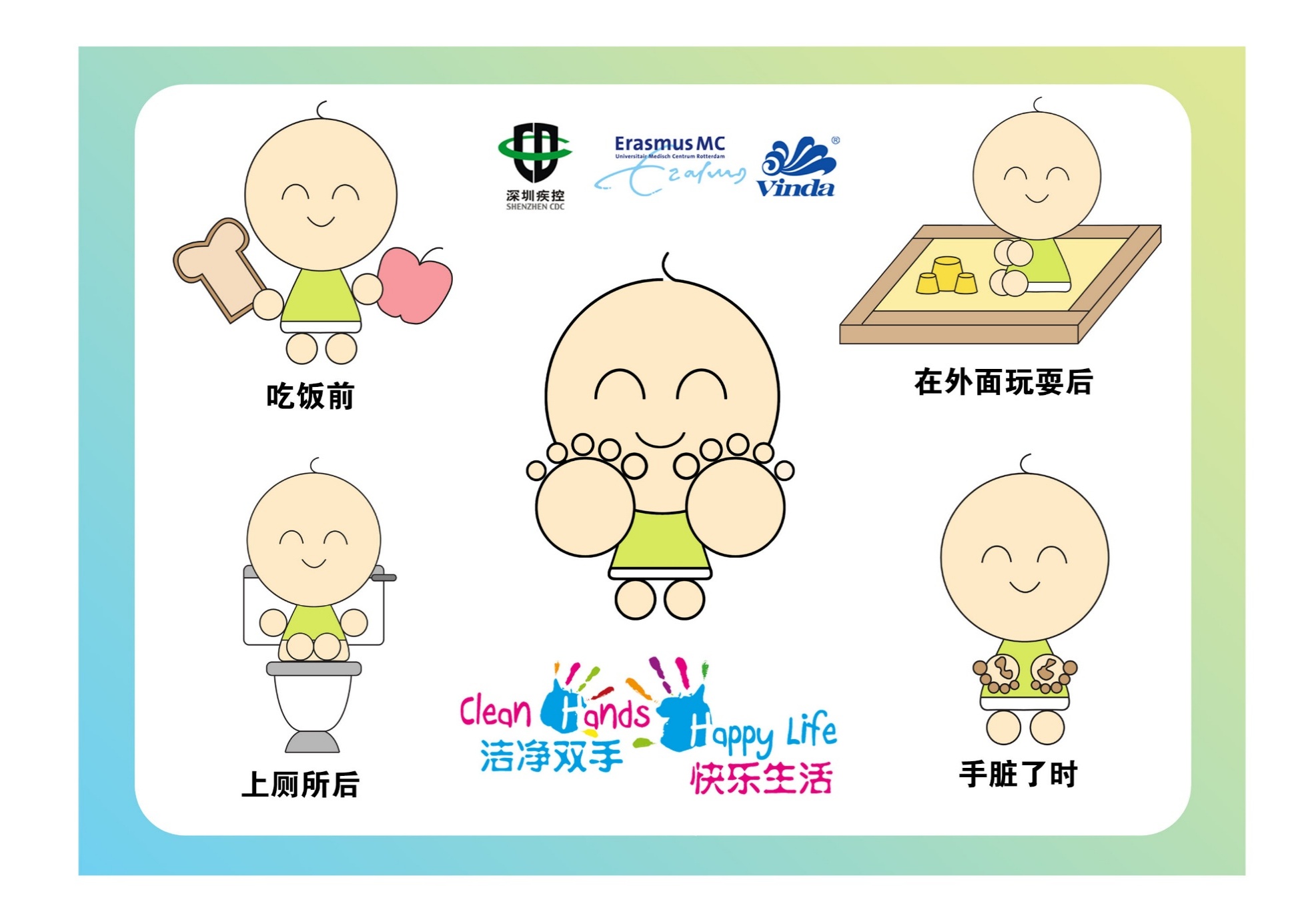
**

**2. 海报 - 儿童洗手步骤图**

**
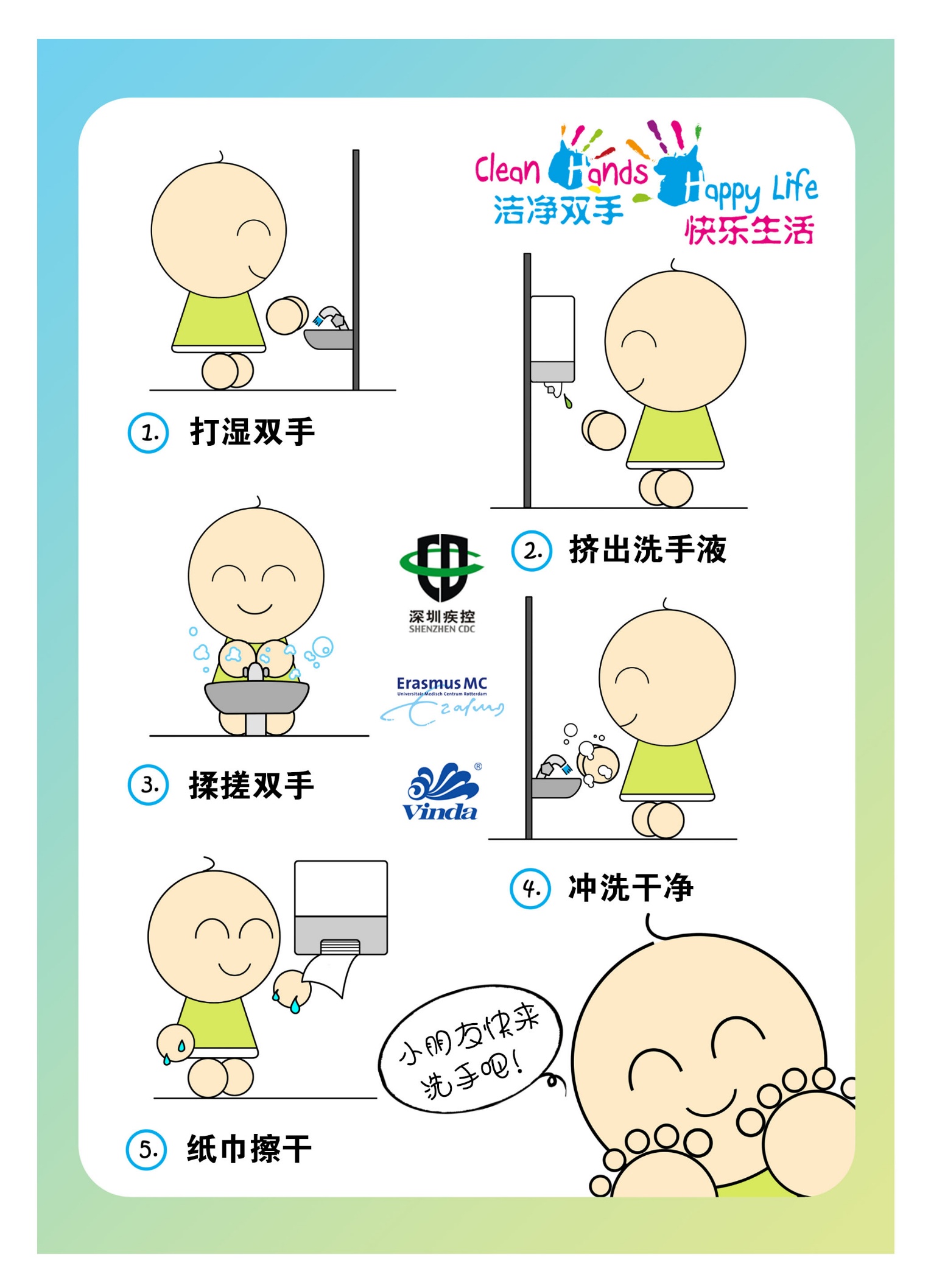
**

**3. 海报 – 何时要洗手（成年人版）**

**
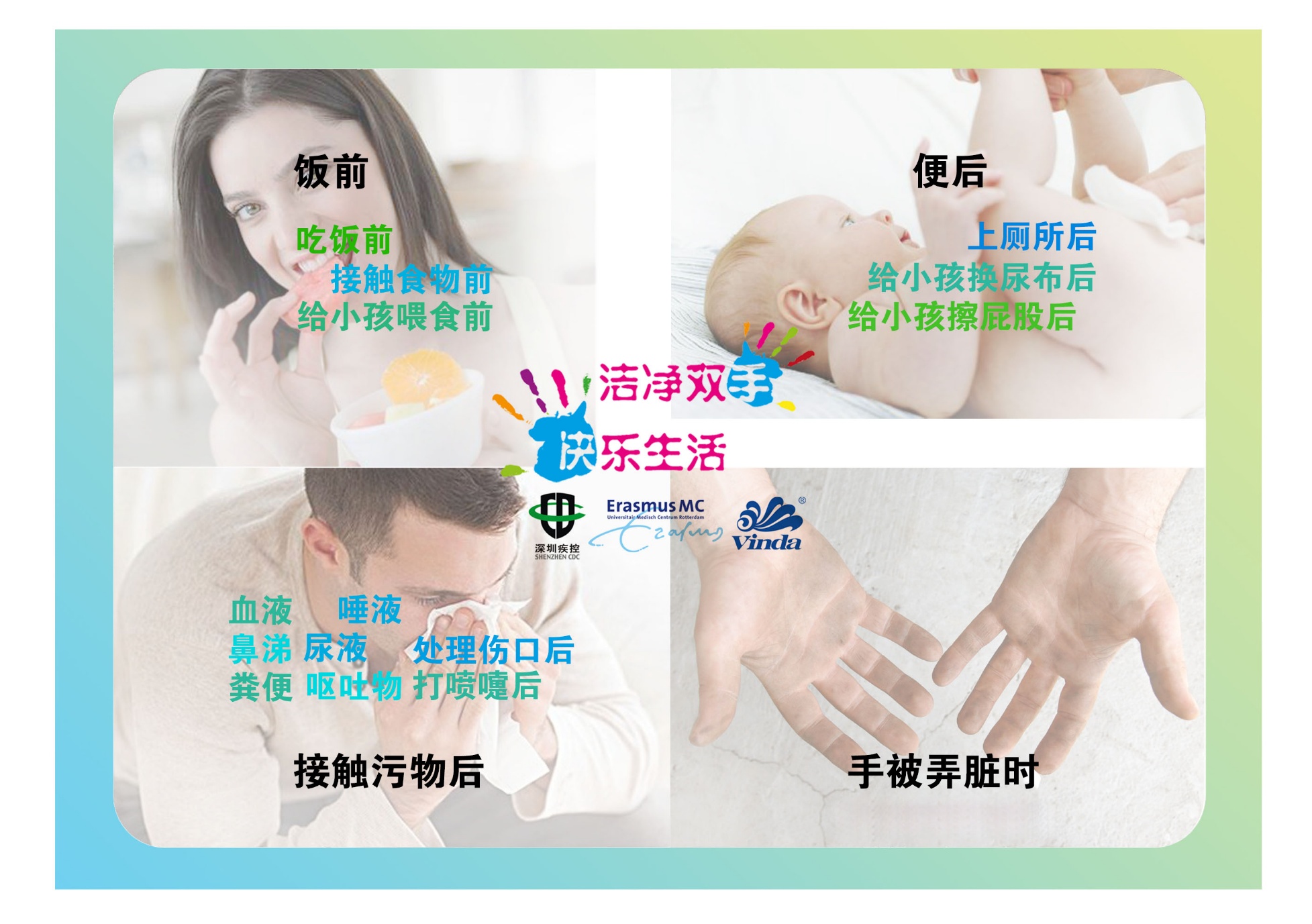
**

**4. 海报 – 成年人洗手步骤图**

**
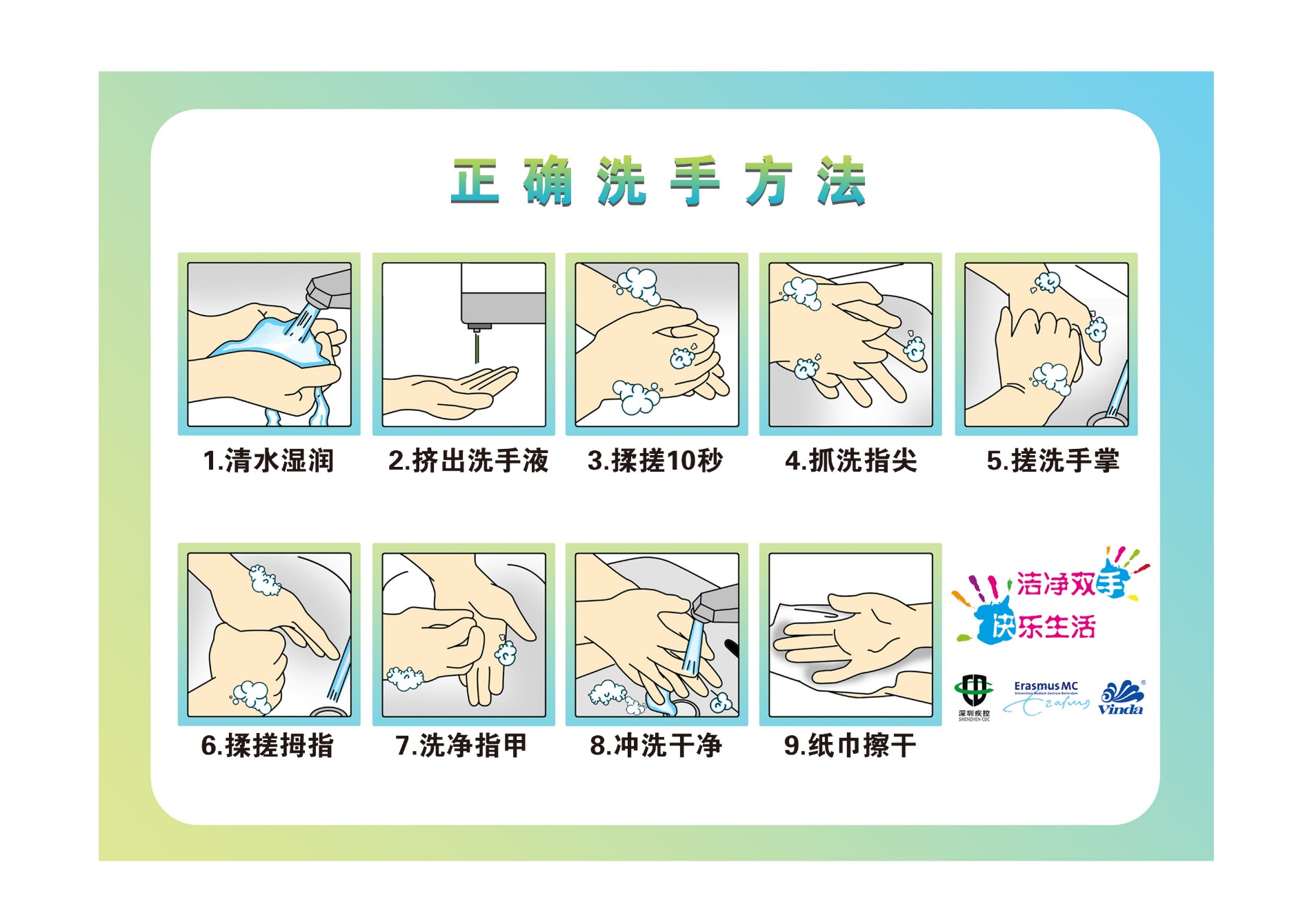
**

**5. 手部卫生知识手册**

**
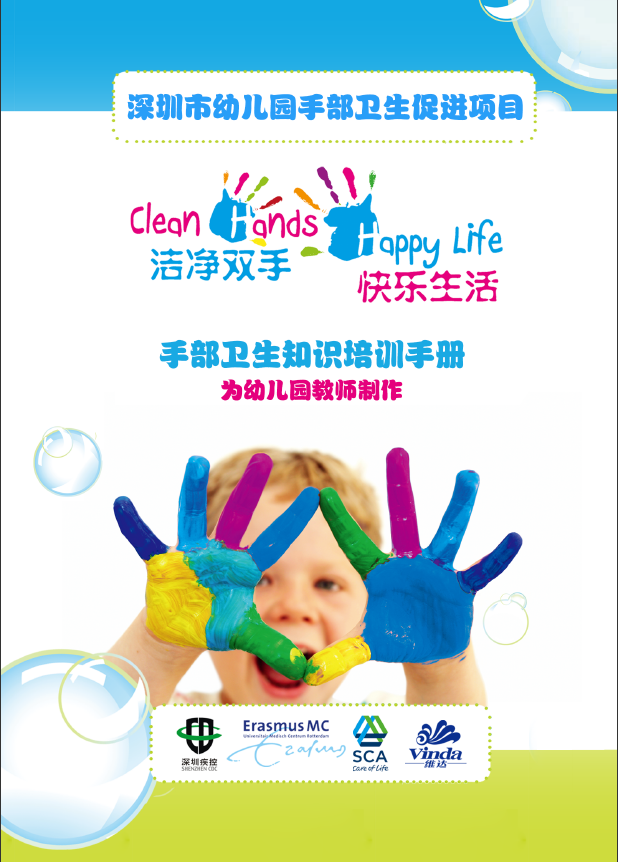
**

**7. Ella和小伙伴们洗手奇遇记（画册）**

**8. Ella洗手学园（手部卫生知识手册）**

**9. Ella洗手学园毕业证**

**10. 手部卫生知识折页**
