# Supplementary material for: A multimodal intervention to improve hand hygiene compliance via social cognitive influences among kindergarten teachers in China
Source: PLoS One. 2019 May 14;14(5):e0215824. doi: 10.1371/journal.pone.0215824 (PMC6516664; doi:10.1371/journal.pone.0215824)
Supplement: S3 File — (DOCX) [file pone.0215824.s003.docx]

编号：

**深圳市幼儿园教师手部卫生情况调查问卷**

尊敬的老师：

您好！为了解幼儿园教师在幼儿园工作时间的洗手情况，我中心特开展此次调查，以制订对策促进幼儿园手部卫生，并预防手部相关传染性疾病。该问卷是匿名填写，所有信息会被严格保密，仅被用于科学研究。且所有问题的答案没有对错之分。请您如实填写！

请注意，洗手指使用肥皂（或洗手液）和水洗手。问卷中的部分条目可能让你感觉是询问相同的问题，但请你仍然填写这些条目。

完成此问卷需耗时10~15分钟左右，如对任何问题有疑问请随时询问我们的调查员。感谢您的支持和配合！

深圳市疾病预防控制中心

**问题范例1（将选项涂黑）：**

|  | 肯定不会 ←→ 肯定会 | | | | | | |
| --- | --- | --- | --- | --- | --- | --- | --- |
| 1．你明天会骑自行车上班的可能性为？ | 1 | 2 | 3 | 4 | 5 | **6** | 7 |

**问题范例2（在选项后打勾）**

2. 你明天会骑自行车上班吗？ （1）不会√ （2）会

**一．个人基本情况**

1.1 您的性别: (1)男 (2)女

1.2您的年龄：__________ 岁

1.3您在幼儿园工作有多少年了？（不仅限于本幼儿园）__________年

1.4您的学历: (1) 初中及以下 (2)高中/中专 (3)大专 (4)本科 (5)研究生以上

1.5您家中是否有14岁以下儿童和您一起居住？ (1)无 (2)有, _____名

1.6平常您的手是否干燥？ (1)从不干燥 (2)有时干燥 (3)一直干燥

1.7您的手是否有湿疹？ (1)从没有 (2)有时会有 (3)一直有

**二．洗手情况**

2.1请描述在各种情况下，您班级儿童和您自己的洗手频率。频率幅度从0（从不洗手）到10（总是会洗手）。在相应数字上打勾。

|  | 从不 ←→ 总是 | | | | | | | | | | |
| --- | --- | --- | --- | --- | --- | --- | --- | --- | --- | --- | --- |
| **下列情况您班上儿童洗手的频率** | | | | | | | | | | | |
| 1. 吃饭前 | 0 | 1 | 2 | 3 | 4 | 5 | 6 | 7 | 8 | 9 | 10 |
| 2. 上厕所后 | 0 | 1 | 2 | 3 | 4 | 5 | 6 | 7 | 8 | 9 | 10 |
| **下列情况您自己洗手的频率** | | | | | | | | | | | |
| 3. 在准备午餐之前 | 0 | 1 | 2 | 3 | 4 | 5 | 6 | 7 | 8 | 9 | 10 |
| 4. 给水果削皮之前 | 0 | 1 | 2 | 3 | 4 | 5 | 6 | 7 | 8 | 9 | 10 |
| 5. 用手捂嘴咳嗽或打喷嚏之后 | 0 | 1 | 2 | 3 | 4 | 5 | 6 | 7 | 8 | 9 | 10 |
| 6. 擦鼻涕之后 | 0 | 1 | 2 | 3 | 4 | 5 | 6 | 7 | 8 | 9 | 10 |
| 7. 换尿布之后 | 0 | 1 | 2 | 3 | 4 | 5 | 6 | 7 | 8 | 9 | 10 |
| 8. 接触体液之后（如唾液，呕吐物，血液，伤口，尿液，鼻涕） | 0 | 1 | 2 | 3 | 4 | 5 | 6 | 7 | 8 | 9 | 10 |
| 9. 户外活动之后 | 0 | 1 | 2 | 3 | 4 | 5 | 6 | 7 | 8 | 9 | 10 |
| 10. 接触脏衣服或脏毛巾之后 | 0 | 1 | 2 | 3 | 4 | 5 | 6 | 7 | 8 | 9 | 10 |
| 11. 上厕所之后 | 0 | 1 | 2 | 3 | 4 | 5 | 6 | 7 | 8 | 9 | 10 |
| 12. 吃饭之前 | 0 | 1 | 2 | 3 | 4 | 5 | 6 | 7 | 8 | 9 | 10 |
| 13. 给儿童喂食之前 | 0 | 1 | 2 | 3 | 4 | 5 | 6 | 7 | 8 | 9 | 10 |
| 14. 给儿童擦鼻涕之后 | 0 | 1 | 2 | 3 | 4 | 5 | 6 | 7 | 8 | 9 | 10 |
| 15. 给儿童擦屁股之后 | 0 | 1 | 2 | 3 | 4 | 5 | 6 | 7 | 8 | 9 | 10 |

2.2 请标出对以下陈述句的同意程度。可选同意程度从1（完全不同意）到7（完全同意）

|  | 完全不同意 ←→ 完全同意 | | | | | | |
| --- | --- | --- | --- | --- | --- | --- | --- |
| 16. 我非常清楚何时应该洗手以及怎样洗手 | 1 | 2 | 3 | 4 | 5 | 6 | 7 |

2.3请判断如下说法是否正确

| 17. 在为儿童准备午餐前必须洗手 | 错误 | 正确 |
| --- | --- | --- |
| 18. 洗手时并不是每次都必须使用肥皂 | 错误 | 正确 |
| 19. 给小孩喂食前应该洗手 | 错误 | 正确 |
| 20. 擦鼻涕后不洗手也没关系 | 错误 | 正确 |

2.4请判断以下情况是否可能：

|  | 不可能 ←→ 非常可能 | | | | | | | | | | |
| --- | --- | --- | --- | --- | --- | --- | --- | --- | --- | --- | --- |
| 21. 你工作的幼儿园有小孩得传染病（例如腹泻，普通感冒） | 0 | 1 | 2 | 3 | 4 | 5 | 6 | 7 | 8 | 9 | 10 |
| 22. 因为你不洗手，你班上的一名儿童得了传染病 | 0 | 1 | 2 | 3 | 4 | 5 | 6 | 7 | 8 | 9 | 10 |
| 23. 因为你的同事不洗手，你班上的一名儿童得了传染病 | 0 | 1 | 2 | 3 | 4 | 5 | 6 | 7 | 8 | 9 | 10 |

2.5您认为以下情况是否严重：

|  | 不严重 ←→ 非常严重 | | | | | | | | | | |
| --- | --- | --- | --- | --- | --- | --- | --- | --- | --- | --- | --- |
| 28. 一名儿童得了传染病 | 0 | 1 | 2 | 3 | 4 | 5 | 6 | 7 | 8 | 9 | 10 |
| 29. 您自己感染了传染病 |  |  |  |  |  |  |  |  |  |  |  |

2.6 您是否每次需要洗手时都会洗手：

|  | 从来不洗 ←→ 每次都洗 | | | | | | | | | | |
| --- | --- | --- | --- | --- | --- | --- | --- | --- | --- | --- | --- |
| 30. 所有需要洗手的情况下我都会洗手 | 0 | 1 | 2 | 3 | 4 | 5 | 6 | 7 | 8 | 9 | 10 |
| 31. 当我很忙时每次也会洗手 | 0 | 1 | 2 | 3 | 4 | 5 | 6 | 7 | 8 | 9 | 10 |
| 32. 即使当发生一些突发事件时，比如接电话，孩子争吵等，每次我仍然会洗手 | 0 | 1 | 2 | 3 | 4 | 5 | 6 | 7 | 8 | 9 | 10 |
| 33.当有传染病发生时，每次我都会洗手 | 0 | 1 | 2 | 3 | 4 | 5 | 6 | 7 | 8 | 9 | 10 |

2.7 您认为您的洗手情况：

|  | 次数更少 ←→ 更加频繁 | | | | | | | | | | |
| --- | --- | --- | --- | --- | --- | --- | --- | --- | --- | --- | --- |
| 34. 与您的同事相比 | 0 | 1 | 2 | 3 | 4 | 5 | 6 | 7 | 8 | 9 | 10 |

**三、洗手规范**

以下问题询问您对“深圳市幼儿园手部卫生促进项目”所提供的洗手规范的看法：

3.1 您是如何看待洗手规范的？

1. 完全不重要 < 1 2 3 4 5 6 7 > 非常重要

2. 很舒适 < 1 2 3 4 5 6 7 > 很不舒适

3. 并未给我洁净清新的感觉 < 1 2 3 4 5 6 7 > 给我洁净清新的感觉

4. 很容易做到 < 1 2 3 4 5 6 7 > 很难做到

5. 会导致手干燥开裂 < 1 2 3 4 5 6 7 > 能避免手干燥开裂

3.2 请回答如下问题：

|  | 肯定不会 ←→ 肯定会 | | | | | | |
| --- | --- | --- | --- | --- | --- | --- | --- |
| 6. 按照规范洗手会花费您过多的时间吗？ | 1 | 2 | 3 | 4 | 5 | 6 | 7 |
| 7. 教室内贴有洗手规范会让您觉得更卫生吗？ | 1 | 2 | 3 | 4 | 5 | 6 | 7 |

3.3 您会按照规范洗手是因为:

|  | 完全不同意 ←→ 完全同意 | | | | | | |
| --- | --- | --- | --- | --- | --- | --- | --- |
| 8. 我班上的儿童会减少感染疾病的风险（比如腹泻，普通感冒等） | 1 | 2 | 3 | 4 | 5 | 6 | 7 |
| 9. 我自己感染传染病的风险会变小 | 1 | 2 | 3 | 4 | 5 | 6 | 7 |

3.4 请标出您对以下说法的同意程度:

|  | 完全不同意 ←→ 完全同意 | | | | | | |
| --- | --- | --- | --- | --- | --- | --- | --- |
| 10. 洗手只能减少细菌传播，但并不能减少病毒传播 | 1 | 2 | 3 | 4 | 5 | 6 | 7 |
| 11. 我并不是经常洗手，这也没有太大关系，因为这会增强儿童的抵抗力 | 1 | 2 | 3 | 4 | 5 | 6 | 7 |
| 12. 我不洗手的话会对儿童带来不良影响 | 1 | 2 | 3 | 4 | 5 | 6 | 7 |

**四．洗手行为**

4.1当面临如下情况是，您是否会按照规范洗手？

|  | 肯定不会 ←→ 肯定会 | | | | | | |
| --- | --- | --- | --- | --- | --- | --- | --- |
| 1. 需要洗手的所有时刻 | 1 | 2 | 3 | 4 | 5 | 6 | 7 |
| 2. 工作压力很大的时候 | 1 | 2 | 3 | 4 | 5 | 6 | 7 |
| 3. 当你手部干燥开裂的时候 | 1 | 2 | 3 | 4 | 5 | 6 | 7 |
| 4. 当你的同事都在洗手的时候 | 1 | 2 | 3 | 4 | 5 | 6 | 7 |

4.2在以后

|  | 完全不同意←→ 完全同意 | | | | | | |
| --- | --- | --- | --- | --- | --- | --- | --- |
| 5. 我计划在任何需要洗手的情况下按照规范洗手 | 1 | 2 | 3 | 4 | 5 | 6 | 7 |
| 6. 我将在任何需要洗手的情况下按照规范洗手 | 1 | 2 | 3 | 4 | 5 | 6 | 7 |

4.3按照规范去洗手：

|  | 完全不同意 ←→ 完全同意 | | | | | | |
| --- | --- | --- | --- | --- | --- | --- | --- |
| 7. 是我自然能做到的事 | 1 | 2 | 3 | 4 | 5 | 6 | 7 |
| 8. 是我自觉能做到的事 | 1 | 2 | 3 | 4 | 5 | 6 | 7 |
| 9. 是我需要努力去做到的事 | 1 | 2 | 3 | 4 | 5 | 6 | 7 |
| 10. 是我日常工作和生活的一部分 | 1 | 2 | 3 | 4 | 5 | 6 | 7 |
| 11. 是我不加思考能做到的事 | 1 | 2 | 3 | 4 | 5 | 6 | 7 |
| 12. 是我本来就一直做的事 | 1 | 2 | 3 | 4 | 5 | 6 | 7 |
| 13. 是我会做因为本就应当做的事 | 1 | 2 | 3 | 4 | 5 | 6 | 7 |

4.4你是如何看待鼓励儿童洗手的

|  | 很困难 ←→ 很容易 | | | | | | |
| --- | --- | --- | --- | --- | --- | --- | --- |
| 14. 我认为鼓励儿童洗手 | 1 | 2 | 3 | 4 | 5 | 6 | 7 |
| 15. 当父母并不重视时，我认为鼓励儿童洗手 | 1 | 2 | 3 | 4 | 5 | 6 | 7 |

**五．你所在幼儿园情况**

这部分关于您工作的幼儿园的一系列情况。请标出您对以下说法的同意程度。

|  | 完全不同意 ←→ 完全同意 | | | | | | |
| --- | --- | --- | --- | --- | --- | --- | --- |
| 1. 我们园长认为我必须按照规范经常洗手 | 1 | 2 | 3 | 4 | 5 | 6 | 7 |
| 2. 我的两名同事认为我应当按照规范经常洗手 | 1 | 2 | 3 | 4 | 5 | 6 | 7 |
| 3. 当我同事按照规范洗手时我感到有压力 | 1 | 2 | 3 | 4 | 5 | 6 | 7 |
| 4. 我的同事都按照规范洗手 | 1 | 2 | 3 | 4 | 5 | 6 | 7 |
| 5 我很重视我同事对按照规范洗手的看法 | 1 | 2 | 3 | 4 | 5 | 6 | 7 |
| 6. 如果我的同事不按照规范洗手，我也不洗 | 1 | 2 | 3 | 4 | 5 | 6 | 7 |
| 7. 洗手是每个人的责任 | 1 | 2 | 3 | 4 | 5 | 6 | 7 |
| 8. 我们幼儿园已有洗手所需全部的材料和设备 | 1 | 2 | 3 | 4 | 5 | 6 | 7 |
| 9. 洗手后我用毛巾擦手，而不是用纸巾 | 1 | 2 | 3 | 4 | 5 | 6 | 7 |
| 10. 用酒精洗手器洗手与肥皂和水一样有效 | 1 | 2 | 3 | 4 | 5 | 6 | 7 |

**您对本问卷和洗手还有其他意见或建议吗？请写下你的看法：**

调查员签名：

调查日期：
