# Supplementary material for: A multimodal intervention to improve hand hygiene compliance via social cognitive influences among kindergarten teachers in China
Source: PLoS One. 2019 May 14;14(5):e0215824. doi: 10.1371/journal.pone.0215824 (PMC6516664; doi:10.1371/journal.pone.0215824)
Supplement: S4 File — (DOCX) [file pone.0215824.s004.docx]

***"Clean Hands, Happy life"***

***Hand hygiene research at china’s kindergartens***

Questionnaire

**Introduction**

The rules are the moments when hand washing should be done. The questions are about time to wash hands working on a group. By hand washing we mean washing hands with soap and water.

Answer the questions based on your instant impression; you do not have to think long. There are no right or wrong answers. We are particularly interested in your opinion on how it goes in practice and the things you encounter. We can use this information to develop measures making it easier for everyone to wash hands frequently. Sometimes, it may seem that a question is repeatedly asked. For the study, it is important that all questions are answered.

This questionnaire is anonymous and strictly confidential.

Completing the questionnaire takes about 10 to 15 minutes. Thank you very much!

**Examples**

You answer the asked question by ticking the corresponding number.

Example:

Certainly not ←→ Certainly

1. Do you think you will go to work tomorrow by bike? 1 2 3 4 5 6 7

2. You go to work by bike. Incorrect Correct

**A. Personal information**

1 What is your gender? 1. man 2. woman

2 What is your age? _____ year

3 How long have you worked as a teaching assistant / teacher since gaining your degree? ____ year

4 What is your educational background? 1 Junior high school or bleow 2 senior high school 3 Junior college 4 Bachelor 5 Master or Doctor

5 Do you have children living at home? 1 No 2 Yes

How many children? _____

6 Do you suffer from dry hands? 1 Never 2 Sometimes 3 Always

7 Do you suffer from eczema? 1 Never 2 Sometimes 3 Always

**B. Rules for hand washing**

The next questions are about how often your children points to the hand washing and how often you wash yourself hands in different situations at the daycare center. What matters is how often it happens in practice. Answer the questions on a scale from 0 (never) to 10 (always), by ticking the corresponding number.

|  | Never ←→ Always | | | | | | | | | | |
| --- | --- | --- | --- | --- | --- | --- | --- | --- | --- | --- | --- |
| How often wise to wash the children's hands ... | | | | | | | | | | | |
| 1. Before eating | 0 | 1 | 2 | 3 | 4 | 5 | 6 | 7 | 8 | 9 | 10 |
| 2. After using toilet |  |  |  |  |  |  |  |  |  |  |  |
| How often was your self your hands ... | | | | | | | | | | | |
| 3. Before the preparation of the lunch | 0 | 1 | 2 | 3 | 4 | 5 | 6 | 7 | 8 | 9 | 10 |
| 4. Before peeling of fruit |  |  |  |  |  |  |  |  |  |  |  |
| 5. After coughing in the hands and / or sneezing |  |  |  |  |  |  |  |  |  |  |  |
| 6. After blowing your nose |  |  |  |  |  |  |  |  |  |  |  |
| 7. After changing a diaper poop |  |  |  |  |  |  |  |  |  |  |  |
| 8. After changing an incontinence diaper when the child is changed on a changing table |  |  |  |  |  |  |  |  |  |  |  |
| 9. After changing an incontinence diaper when the child is standing changed |  |  |  |  |  |  |  |  |  |  |  |
| 10. After contact with body fluids  (saliva, vomit, blood, wound, urine, snot) |  |  |  |  |  |  |  |  |  |  |  |
| 11. After playing outside |  |  |  |  |  |  |  |  |  |  |  |
| 12. After contact with soiled textiles (dirty washcloths, bibs, burp cloths, towels) |  |  |  |  |  |  |  |  |  |  |  |
| 13. After going to the toilet |  |  |  |  |  |  |  |  |  |  |  |
| 15. Before you go eat yourself |  |  |  |  |  |  |  |  |  |  |  |
| 16. Before you help a child with food |  |  |  |  |  |  |  |  |  |  |  |
| 17. After wiping a nose of a child after |  |  |  |  |  |  |  |  |  |  |  |
| 18. After butt wiping a child |  |  |  |  |  |  |  |  |  |  |  |

Answer the following statement on a scale from 1 (definitely not) to 7 (certainly):

|  | Certainly not ←→ Certainly | | | | | | |
| --- | --- | --- | --- | --- | --- | --- | --- |
| 19. I know exactly what the rules are for washing hands. | 1 | 2 | 3 | 4 | 5 | 6 | 7 |

Give to the following statements always on whether they are right or wrong, by ticking the appropriate box. The guidelines state:

|  | Incorrect | Correct |
| --- | --- | --- |
| 20. Before the preparation of the lunch, the hands must be washed. |  |  |
| 21. Hands should be washed after changing every pee diaper. |  |  |
| 22. By washing hands, it is not always necessary to use soap. |  |  |
| 23. Before you help children to eat, the hands should be washed. |  |  |
| 24. After blowing your nose, the hands are ok not washed. |  |  |

What the possibility is that....

|  | Very small ←→ Very big | | | | | | | | | | |
| --- | --- | --- | --- | --- | --- | --- | --- | --- | --- | --- | --- |
| 25.An infectious disease (such as diarrhea, common cold) occurs in your child day center? | 0 | 1 | 2 | 3 | 4 | 5 | 6 | 7 | 8 | 9 | 10 |
| 26.A child in your group infected because you do not wash your hands? |  |  |  |  |  |  |  |  |  |  |  |
| 27.A child in your group infected because your colleagues do not wash their hands? |  |  |  |  |  |  |  |  |  |  |  |

How serious are the following possibilities…

|  | Not serious ←→ Very serious | | | | | | | | | | |
| --- | --- | --- | --- | --- | --- | --- | --- | --- | --- | --- | --- |
| 28. For a child as he or she get an infection | 0 | 1 | 2 | 3 | 4 | 5 | 6 | 7 | 8 | 9 | 10 |
| 29. For yourself as you get an infection |  |  |  |  |  |  |  |  |  |  |  |

To what extent do you wash your hands ...

|  | Never ←→ Always | | | | | | | | | | |
| --- | --- | --- | --- | --- | --- | --- | --- | --- | --- | --- | --- |
| 30. In all situations where it is needed? | 0 | 1 | 2 | 3 | 4 | 5 | 6 | 7 | 8 | 9 | 10 |
| 31. When is busy? |  |  |  |  |  |  |  |  |  |  |  |
| 32. If you are distracted by other events such as telephone, quarrel between the children, etc? |  |  |  |  |  |  |  |  |  |  |  |
| 33. As there is something prevails |  |  |  |  |  |  |  |  |  |  |  |

To what extent do you wash your hands ...

|  | Much less often ←→ Much more often | | | | | | | | | | |
| --- | --- | --- | --- | --- | --- | --- | --- | --- | --- | --- | --- |
| 34. Compared to your peers? | 0 | 1 | 2 | 3 | 4 | 5 | 6 | 7 | 8 | 9 | 10 |

***End***

Do you have any comments or suggestions about the questionnaire and / or washing hands in practice? We'd love to hear them!

_________________________________________________________________

***Thank you for your participation!***
