# Supplementary material for: A multimodal intervention to improve hand hygiene compliance via social cognitive influences among kindergarten teachers in China
Source: PLoS One. 2019 May 14;14(5):e0215824. doi: 10.1371/journal.pone.0215824 (PMC6516664; doi:10.1371/journal.pone.0215824)
Supplement: S6 File — (PDF) [file pone.0215824.s006.pdf]

Journal of Infection 000 (2018) 1–8

Contents lists available at ScienceDirect

Journal of Infection

journal homepage: www.elsevier.com/locate/jinf

# A hand hygiene intervention to decrease hand, foot and mouth disease and absence due to sickness among kindergarteners in China: A cluster-randomized controlled trial

Xiaona Liu[a,b,1,*], Wanli Hou[a,1], Zhiguang Zhao[a], Jinquan Cheng[a,**], Ed F. van Beeck[b], Xiaodong Peng[a], Kylah Jones[c], Xia Fu[a], Yan Zhou[a], Zhen Zhang[a], Jan Hendrik Richardus[b], Vicki Erasmus[b]

[a] *Shenzhen Center for Disease Control and Prevention, Longyuan Road No.8, Nanshan District, Shenzhen 518055, China*
[b] *Erasmus MC, University Medical Centre Rotterdam, Dr. Molewaterplein 40, 3015 GD Rotterdam, The Netherlands*
[c] *Athena Institute, Vrije Universiteit Amsterdam, Amsterdam, The Netherlands*

ARTICLE INFO

*Article history:*
Accepted 14 August 2018
Available online xxx

*Keywords:*
Kindergarten
Hand Hygiene
Infectious disease control
Youth health
Community-based intervention

SUMMARY

*Objectives:* : To evaluate the effect of the "Clean Hands, Happy Life" intervention on the incidence of hand, food and mouth disease (HFMD) and on school absences due to sickness in kindergarten students.
*Methods:* : The intervention consisted of four hand hygiene (HH) promotion components and was evaluated in a cluster-randomized controlled trial among 8275 children and 18 kindergartens from May to October, 2015 in Shenzhen, China. We compared two intervention arms – received the intervention in kindergartens only and in both kindergartens and families, respectively – to the control arm in multilevel analyses.
*Results:* : During the follow-up, the incidence of HFMD in both intervention arms was significantly lower than in the control arm ($IRR_1$: 0.39, 95%CI: 0.26–0.59; $IRR_2$: 0.30, 95%CI: 0.19–0.49); the duration of absence due to sickness (in days) in both intervention arms was significantly shorter than in the control arm ($\beta_1 = 0.58$, 95%CI: 0.41–0.74; $\beta_2 = 0.34$, 95%CI: 0.17–0.50), controlling for the area type of kindergarten and grade level of children. Furthermore, during the follow-up we found that there were fewer episodes of absence due to respiratory, skin and eye infections ($P < 0.05$).
*Conclusions:* : Our intervention is effective at reducing HFMD infections and absence due to sickness in children attending kindergartens in China.
© 2018 The British Infection Association. Published by Elsevier Ltd. All rights reserved.

## Introduction

Hand, foot and mouth disease (HFMD) has recently emerged in the Asia-Pacific region as the most severe epidemic disease affecting infants and young children.[1] Children younger than five years old are especially susceptible to HFMD, as they do not yet have protective immunity to Enterovirus 71 (EV71) or Coxsackievirus virus A 16 (CV-A16), which are the common viral pathogens that lead to HFMD.[2] Asymptomatic adult carriers of EV71 and CA16 are a major reservoir for the transmission of these viruses.[3] HFMD transmission can be caused by contact with the nasal and oral discharge of HFMD patients, or by touching skin, hands, herpes fluid or other contaminated items.[4] Most infected children display mild and self-limiting illness typically including fever, skin eruptions on hands and feet, and vesicles in the mouth. However, some children rapidly develop neurological, cardiovascular and respiratory complications that can be fatal.[5]

In China, HFMD has been designated as a notifiable disease and should be reported through a national surveillance system which was established in response to several large outbreaks in 2007 and early 2008.[6] The national surveillance registry system documented over seven million probable cases between 2008 and 2012, of which 3.7% were laboratory confirmed and 0.03% were fatal.[7] In China, children attending kindergartens are generally between the ages of three and six years. Children in this age group are vulnerable to HFMD infection. Moreover, many kindergartens have over 20 children in a single classroom; where stagnant air and

* Corresponding author at: Erasmus MC, University Medical Centre Rotterdam, Dr. Molewaterplein 40, 3015 GD Rotterdam, The Netherlands.
** Corresponding author. Shenzhen Center for Disease Control and Prevention, Longyuan Road No.8, Nanshan District, Shenzhen 518055, China.
*E-mail addresses:* x.liu@erasmusmc.nl (X. Liu), cjinquan@szcdc.net (J. Cheng).
[1] Wanli Hou and Xiaona Liu contributed equally to this manuscript.

https://doi.org/10.1016/j.jinf.2018.08.009
0163-4453/© 2018 The British Infection Association. Published by Elsevier Ltd. All rights reserved.

Please cite this article as: X. Liu et al., A hand hygiene intervention to decrease hand, foot and mouth disease and absence due to sickness among kindergarteners in China: A cluster-randomized controlled trial, Journal of Infection (2018), https://doi.org/10.1016/j.jinf.2018.08.009

close contact between children assists the transmission of HFMD pathogens.[8]

Although kindergartens face risks of being shut down due to identification of clusters of five or more cases of HFMD cases within a week, which is regulated by China's national guideline on public health response for HFMD aggregation and outbreak,[9] very few of them provide screening or prevention programs for HFMD partially because of the combination of limited school doctors and a large number of children.[10] Also, severe HFMD is now vaccine-preventable in China, as China's Food and Drug Administration approved the world's first vaccine against EV71[11] in 2016. However, it is not included in the national vaccination program. Practicing good hand hygiene (HH) has been universally recognized as a common strategy to decrease the risk of infection with the viruses that cause HFMD.[12] Nevertheless, the HH compliance of children is sub-optimal all over the world. A study in Vietnam suggests that only 11.5% of the children washed their hands after using the toilet.[13] Even in the relatively wealthy countries, such as the Netherlands, studies have shown children's overall compliance is around 38%.[14]. In China, the HH compliance of students in primary schools has been found to be around 20%.[15,16]

Previously, a HH intervention was developed in the Netherlands and implemented in Dutch day care centres, successfully improving HH compliance among both children and care givers.[17] In response to growing concerns about HFMD infections in kindergartens in China, we developed a culturally-tailored intervention based on the Dutch intervention and carried it out in kindergartens in the city of Shenzhen to improve the HH compliance of kindergarten children and teachers, and eventually prevent HFMD. In previous years, a seasonal peak of HFMD cases was observed between May and July, as well as a smaller peak between September and October annually in Shenzhen.[10] To cover both seasonal peaks, we planned the implementation of the intervention from May to October.

This is a part of a larger study on evaluating our developed intervention, aiming to report the effects of the intervention on the incidence of HFMD among children attending kindergartens in China. Additionally, this study aimed to evaluate the possible effect of the intervention on other diseases that had also been recognized as being preventable by good hand-washing practices. The study also assessed the effect on the children's school absence due to sickness in general.

## Materials and methods

### *Study design and sample size*

A HH intervention was tested by a cluster-randomized controlled trial with kindergartens as the unit of randomization in Shenzhen between May and October, 2015 (Fig. 1). Using the methods recommended by Breukelen and Candel[18] we calculated sample size taking into account the intracluster correlation coefficient (ICC), the cost per cluster and cost per person, the expected effect on behaviour change, the power and the significance level of the study. We assumed an ICC of 0.1 adjusted from a previous similar trial in Colombia,[19] and specified the cost per cluster to be 2500 RMB (360 US dollar, targeting budget on purchasing and setting up materials for classroom and family use) and the cost per person to be 10 RMB (1.5 US dollar, targeting budget on printing materials for individual use); the power was 90% and the significance level was 0.05 (two-sided). This resulted in a minimum of 18 clusters with each cluster containing 48 children to detect the expected compliance with HH guideline increased from 30% (baseline data, unpublished) to 80%, which was the primary outcome of the large study (reported in another paper, unpublished).

### *Randomization and masking*

In this trial we used computer generated random allocation to assign 18 kindergartens to one of three arms (two intervention arms, one control arm) to represent three types of kindergartens and two types of local areas (Fig. 1). First, we stratified all districts of Shenzhen based on whether the majority of the district was in an urban or semi-urban area. Among seven districts, the Nanshan district was chosen to represent urban areas, while the Baoan district was chosen to represent semi-urban areas. Second, we clustered all full-time kindergartens into one of the two districts based on the type of kindergarten. China has a vast and varied school system with several types of kindergartens. This study classified full-time kindergartens into one of three types, namely government-owned, privately owned (targeting higher SES), and migrant – a type of privately owned kindergarten specifically targeting children of domestic migrants (generally lower SES) (20). There were 300–800 children in each of the enrolled kindergartens. Their ages ranged from three to six years. All the children in the selected kindergartens were enrolled in this study; parents and kindergarten doctors signed informed consent forms prior to commencement of the study.

### *Intervention program*

Two intervention arms were compared to the control arm in this study. The first intervention arm was delivered for 2865 children in six kindergartens and the second intervention arm was delivered for 2385 children in six kindergartens as well as to their families, while the control arm with 3025 children in six kindergartens continued usual practice but received the intervention after the study (Fig. 1). The intervention consisted of four components which were provided by Tork. First, the following HH products were provided free of charge with refills for six months: soap dispensers, towel dispensers, paper towels and soap. Second, reminders and cues for action were provided free of charge, including posters, stickers, HH-related reading books, memory games, colouring pages and a hand-washing diploma designed for children. Third, one kick-off event was performed in each kindergarten with four stations, including a hand tablet based hygiene e-game (Ella's Handwashing Adventure, Tork), hand-washing instructions, educational story time, and diploma award. Fourth, training was given to educate kindergarten teachers about the national guideline of HH in the first intervention arm, while the second intervention arm added the training for children's parents and/or guardians at home. This included a hand-washing exercise using UV Glow Cream and an information booklet outlining the content of the training, which was based on an HH training session developed in Dutch day care centres.[21] All intervention materials were pre-tested to be appropriate to read or play for the Han Chinese (ethnicity) children from mainland China.[22]

### *Outcome measurement*

Before launching the intervention, a baseline survey was conducted among parents of all children in the participating kindergartens. Parents were asked to provide information on their children's gender, age, medical history, household and parenting status. HFMD incidence rate and absence due to sickness were measured using a self-designed absence card and monitored throughout the intervention by kindergarten doctors who gathered the information either based on their own observation or parents' report, from May 8th to October 30th in 2015. The absence card included questions regarding signs and symptoms of sickness, health-seeking behaviour, absence date and duration, and diagnosis by clinical professionals. We identified 24 diseases based

Please cite this article as: X. Liu et al., A hand hygiene intervention to decrease hand, foot and mouth disease and absence due to sickness among kindergarteners in China: A cluster-randomized controlled trial, Journal of Infection (2018), https://doi.org/10.1016/j.jinf.2018.08.009

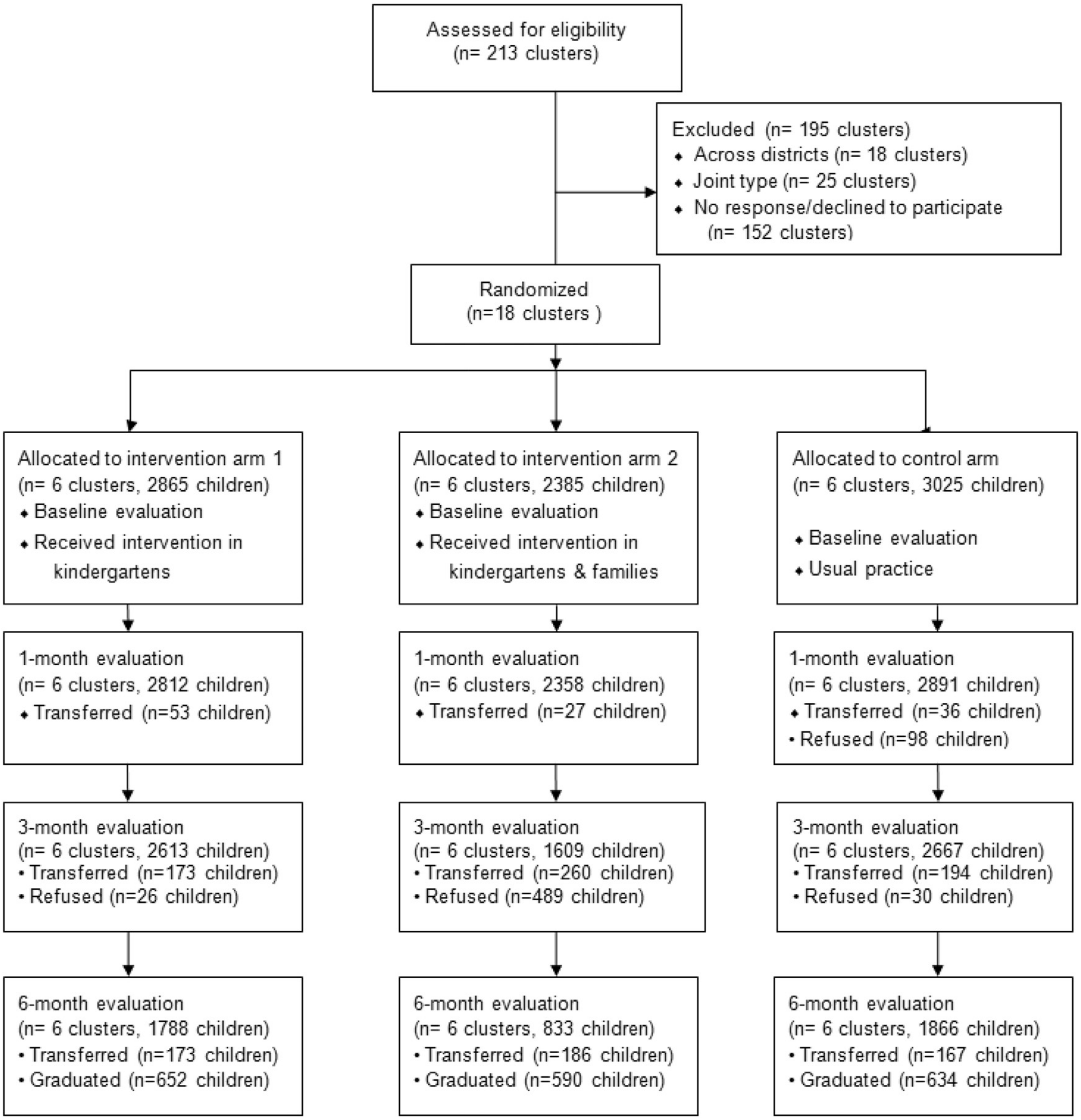


**Fig. 1.** Flow chart for cluster randomized controlled trial comparing a hand hygiene intervention for kindergartens and for both of kindergartens and families with usual practice for kindergartens in Baoan and Nanshan districts, Shenzhen.

on the reported diagnosis, i.e., HFMD, Upper respiratory tract infection, Pharyngitis, Inflammation of the amygdala, Bronchitis, Benign croups angina, Pneumonia, Gastroenteritis, Ophthalmia, Hordeolum, Irritability, Trauma, Indigestion, Varicella, Saprodontia, Ottitis media, Measles, Eczema, Oral ulcer, Asthma, Lymphadenitis, Mumps, Ear periarthritis, and Dermatitis. We also identified absence cases due to common cold and/or diarrhoea based on the reported symptoms. HFMD was defined as presenting a rash of flat discoloured spots and bumps and/or facial ulcers, blisters or lesions in or around the nose or mouth. The common cold was defined as a blocked or runny nose with at least one of the following symptoms: coughing, sneezing, fever, sore throat, or earache. Diarrhoea was defined as two or more watery or unusually loose stools within 24 h. If the diagnosis was unclear, a throat swab or stool specimen was taken to identity the virus. Based on the International Statistical Classification of Diseases and Related Health Problems 10th Revision (ICD-10),[23] we categorized Cold; Upper respiratory tract infection; Pharyngitis; Inflammation of the amygdala; Bronchitis; Benign croups angina; Pneumonia as Respiratory illness; Gastroenteritis and Diarrhoea as Gastrointestinal illness; and Ophthalmia and Hordeolum as Ophthalmitis.

*Data analyses*

All data were entered twice before initiating analysis to ensure the accuracy of all data extracted from the collected absence cards. Data were analyzed using SPSS version 21 (SPSS Inc., USA) and R version 2.12.2 (R Foundation, Austria). Analyses were performed according to the intention-to-treat principle, i.e., including all intervention kindergartens regardless of whether they completed all training programs, used designated posters/sticks/games/books/colour pages or HH products. First, one-way analysis of variance with Chi-Square and analysis of variance (ANOVA) were used to compare baseline characteristics of children after clustering the data by kindergartens. Second, the incidence rate of diseases was calculated during the six-month follow-up period in the two intervention arms and the control arm, after clustering all data by kindergartens. The incidence rate

Please cite this article as: X. Liu et al., A hand hygiene intervention to decrease hand, foot and mouth disease and absence due to sickness among kindergarteners in China: A cluster-randomized controlled trial, Journal of Infection (2018), https://doi.org/10.1016/j.jinf.2018.08.009

**Table 1**
Comparison of baseline characteristics of children among the three arms in the trial, clustered by kindergartens.

| | | Intervention arm 1 $N = 2865$ $n$ (%) | Intervention arm 2 $N = 2385$ $n$ (%) | Control arm $N = 3025$ $n$ (%) | *P* value |
|---|---|---|---|---|---|
| Gender | Boy | 1557 (54.3) | 1306 (54.8) | 1650 (54.5) | 0.989 |
| | Girl | 1308 (34.6) | 1079 (45.2) | 1375 (45.5) | |
| Age | ≤ 4 | 1049 (36.6) | 809 (33.9) | 972 (32.1) | 0.241 |
| | 5 | 855 (29.8) | 732 (30.7) | 1015 (33.6) | |
| | ≥ 6 | 961 (33.5) | 844 (35.4) | 1038 (34.3) | |
| Single parent | Yes vs. no | 73 (2.7) | 56 (2.5) | 71 (2.5) | 0.939 |
| Daily guardian | Parents | 2122 (74.4) | 1756 (73.9) | 2186 (72.7) | 0.845 |
| | Grand parents | 561 (19.7) | 496 (20.9) | 655 (21.8) | |
| | Others | 171 (6.0) | 123 (5.2) | 165 (5.5) | |
| Family monthly income | ≤ 10,000 Yuan | 1001 (36.6) | 777 (33.8) | 939 (32.7) | 0.989 |
| | 10,000–30,000 Yuan | 1265 (46.2) | 1216 (52.8) | 1543 (53.7) | |
| | ≥ 30,000 Yuan | 470 (17.2) | 308 (13.4) | 389 (13.5) | |
| Household registration | Local vs. migration | 1718 (61.2) | 1566 (66.7) | 2192 (73.5) | 0.952 |
| Living area | Urban vs. semi-urban | 1718 (60.0) | 1566 (65.7) | 2192 (72.5) | 0.951 |
| Chronic diseases | Yes vs. no | 204 (7.2) | 207 (8.7) | 257 (8.5) | 0.476 |
| Medicine intake | Yes vs. no | 353 (12.6) | 295 (12.7) | 365 (12.4) | 0.917 |
| Live with sibling(s) | Yes vs. no | 1059 (37.8) | 920 (39.4) | 1015 (34.7) | 0.848 |
| Education level of daily guardian | ≤ Secondary school | 234 (8.2) | 203 (8.6) | 228 (7.7) | 0.916 |
| | High school | 1266 (44.6) | 989 (41.7) | 1207 (40.9) | |
| | ≥ College degree | 1337 (47.1) | 1178 (49.7) | 1515 (51.4) | |

Note: Intervention arm 1 involves the intervention delivered in eight kindergartens; Intervention arm 2 involves the intervention delivered in eight kindergartens and participated children's families.

(IR) was defined as the number of disease episodes ($A$) per child-year ($T$), and an episode was defined as illness diagnosed by clinic professionals or the report of symptoms that meet the definition of illness (i.e., cold and diarrhoea). Episodes of illness which started on the first day that parents and/or school doctors started monitoring disease incidence were excluded. The time at risk was defined as school days during the follow-up period, excluding weekends, national holidays and summer vacation (the month of August). Third, crude incidence rate ratios (IRR) with 95% confidence intervals (CIs) were obtained for all monitored diseases in multilevel Poisson regression analysis with the count of absence due to specific disease as dependent variable, log (child years at risk) included as an offset and adjustment for clustering by kindergartens. IRR was defined as $\mathrm{IRR} = \frac{A_1 \div T_1}{A_0 \div T_0}$, $SD[\ln(\mathrm{IRR})] = \left(\frac{1}{A_1} + \frac{1}{A_0}\right)^{1/2}$, 95%CI (IRR)= exp{ln(IRR)±1.96SD[ln(IRR)]}.[24] Adjusted IRR for HFMD were further obtained in a multivariate Poisson regression model after correcting for variation in follow-up months, area and type of kindergartens. Two-way interactions were examined for evidence of intervention effect modification. Finally, the mean duration of absence due to HFMD was calculated for HFMD-diagnosed children. Multilevel linear regression analyses were performed to compare the effect of the intervention arms on the absence duration for all cases corrected by the area and type of the kindergartens, after clustering the data by kindergartens.

## Results

### *Baseline characteristics of participating children*

There were 8275 children and 18 kindergartens participating in the baseline survey of the trial (Table 1). Most of the children in the study did not suffer from any chronic diseases (>90%) and did not take medication (>85%); they were cared for by their parents (>70%) who were not single (>97%) and had completed at least a high school education (90%). Most of the families had a monthly income of 10,000 to 30,000 Yuan (approximately 1428–4300 US dollars), were residing locally with their household registered in Shenzhen (60%) and were living in urban areas (60%). The comparison of the children's characteristics after clustering indicates that there was no significant difference between the intervention and control arms regarding the children's gender, age, living and parenting status and medical history ($P>$ 0.05). Incidence of absence episodes due to sickness were monitored in 8275 children for 83 days. Of these children, 34 were lost to follow-up in the control arm (1.12%), 30 in the first intervention arm (1.05%) and 29 in the second intervention arm (1.22%) mostly due to school transfer (>95%) (Fig. 1).

### *Effect of the trial on the incidence of HFMD*

Table 2 shows the incidence of absence episodes due to the monitored diseases comparing the two intervention arms with the control arm separately. Within 83 days as the time at risk for monitoring, in total 2911 episodes were reported in the first intervention arm and 3084 episodes in the second intervention arm vs. 3371 episodes in the control arm. Among which, 30 were due to HFMD in the first intervention arm and 22 in the second intervention arm vs.79 in the control arm. After clustering data by kindergartens, the incidence of HFMD was 0.04 episodes per child-year in both intervention arms, which was significantly lower than the incidence of HFMD in the control arm, which was 0.11 episodes per child-year ($IRR_1$: 0.39, 95%CI: 0.26–0.59; $IRR_2$: 0.30, 95%CI: 0.19–0.49). There was no significant difference in the incidence rate of HFMD between the two intervention arms ($P>$ 0.05). The calculated ICC was 0.047.

Fig. 2 illustrates the incidence of absence episodes due to HFMD in the intervention and control arms over time, in different districts and in different types of kindergartens. The incidence of absence episodes in each of the intervention arms was lower than that in the control arm at the first month follow-up (0.10 and 0.07 vs. 0.32 episodes per child-year in May), while at the three-month follow-up it was the same and lowest for both the first intervention arm and control arm. At the six-month follow-up, it was the same again after the control arm increased the incidence between July and October (0.01 episodes per child-year in October). Generally, kindergartens in the Baoan district (semi-urban) and owned by government or privately had a higher incidence than those in the Nanshan district (urban) and targeting domestic migrants. Compared to kindergartens in the control arm, those enrolled in the first intervention arm did not significantly decrease the risk of contact HFMD (IRR: 0.48, 95% CI: 0.17–1.32), while those

**Table 2**
Effect of the trial on incidence of absence episodes due to monitored diseases measured by incidence rate (IR, episodes per child year) and incidence rate ratios (IRR) among children in Shenzhen, China.

| Monitored diseases | Intervention arm 1 | | Intervention arm 2 | | Control arm | | | 95%CI($IRR_1$) | | | 95%CI($IRR_2$) | |
|---|---|---|---|---|---|---|---|---|---|---|---|---|
| | Episodes | $IR_1$ | Episode | $IR_2$ | Episodes | $IR_0$ | $IRR_1$ | Lower | Upper | $IRR_2$ | Lower | Upper |
| **HFMD** | 30 | 0.04 | 22 | 0.04 | 79 | 0.11 | 0.39 | 0.26 | 0.59 | 0.30 | 0.19 | 0.49 |
| **Respiratory illness** | 2081 | 3.19 | 2366 | 4.36 | 2551 | 3.69 | 0.83 | 0.79 | 0.88 | 1.01 | 0.96 | 1.07 |
| Cold | 1396 | 2.21 | 1425 | 2.61 | 1452 | 2.20 | 0.98 | 0.91 | 1.06 | 1.07 | 1.00 | 1.15 |
| Upper respiratory tract infection | 200 | 0.30 | 359 | 0.64 | 430 | 0.55 | 0.48 | 0.40 | 0.56 | 0.91 | 0.79 | 1.05 |
| Pharyngitis | 284 | 0.37 | 288 | 0.56 | 270 | 0.38 | 1.08 | 0.91 | 1.27 | 1.16 | 0.99 | 1.37 |
| Inflammation of the amygdala | 82 | 0.12 | 144 | 0.26 | 164 | 0.24 | 0.51 | 0.39 | 0.67 | 0.96 | 0.77 | 1.20 |
| Bronchitis | 55 | 0.08 | 40 | 0.07 | 143 | 0.20 | 0.39 | 0.29 | 0.54 | 0.31 | 0.22 | 0.43 |
| Benign croups angina | 48 | 0.08 | 98 | 0.19 | 70 | 0.09 | 0.70 | 0.49 | 1.01 | 1.53 | 1.12 | 2.08 |
| Pneumonia | 16 | 0.03 | 12 | 0.02 | 22 | 0.03 | 0.74 | 0.39 | 1.42 | 0.60 | 0.30 | 1.20 |
| **Gastrointestinal illness** | 83 | 0.12 | 100 | 0.19 | 63 | 0.09 | 1.35 | 0.97 | 1.87 | 1.73 | 1.26 | 2.37 |
| Gastroenteritis | 53 | 0.07 | 79 | 0.15 | 55 | 0.08 | 0.98 | 0.68 | 1.44 | 1.57 | 1.11 | 2.21 |
| Diarrhoea | 30 | 0.05 | 21 | 0.04 | 8 | 0.01 | 3.93 | 1.76 | 8.36 | 2.87 | 1.27 | 6.47 |
| **Ophthalmitis** | 14 | 0.02 | 22 | 0.04 | 35 | 0.05 | 0.41 | 0.22 | 0.76 | 0.69 | 0.40 | 1.17 |
| Ophthalmia | 13 | 0.02 | 20 | 0.04 | 28 | 0.03 | 0.49 | 0.25 | 0.96 | 0.91 | 0.50 | 1.63 |
| Hordeolum | 1 | 0.00 | 2 | 0.00 | 7 | 0.01 | 0.15 | 0.02 | 1.18 | 0.31 | 0.07 | 1.50 |
| **Irritability** | 33 | 0.05 | 55 | 0.10 | 47 | 0.06 | 0.72 | 0.46 | 1.12 | 1.28 | 0.87 | 1.89 |
| **Trauma** | 41 | 0.07 | 53 | 0.10 | 44 | 0.06 | 0.95 | 0.62 | 1.46 | 1.32 | 0.88 | 1.96 |
| **Indigestion** | 21 | 0.03 | 39 | 0.07 | 35 | 0.03 | 0.61 | 0.36 | 1.05 | 1.22 | 0.77 | 1.92 |
| **Varicella** | 5 | 0.01 | 2 | 0.00 | 19 | 0.03 | 0.27 | 0.10 | 0.72 | 0.12 | 0.03 | 0.49 |
| **Saprodontia** | 19 | 0.03 | 18 | 0.04 | 18 | 0.03 | 1.08 | 0.57 | 2.01 | 1.09 | 0.57 | 2.10 |
| **Otitis media** | 2 | 0.00 | 7 | 0.01 | 14 | 0.02 | 0.15 | 0.03 | 0.64 | 0.55 | 0.22 | 1.35 |
| **Measles** | 3 | 0.00 | 1 | 0.00 | 13 | 0.01 | 0.24 | 0.07 | 0.83 | 0.09 | 0.01 | 0.64 |
| **Eczema** | 17 | 0.04 | 15 | 0.03 | 13 | 0.02 | 1.34 | 0.65 | 2.75 | 1.26 | 0.60 | 2.65 |
| **Oral ulcer** | 12 | 0.02 | 31 | 0.06 | 9 | 0.01 | 1.36 | 0.57 | 3.23 | 3.76 | 1.79 | 7.90 |
| **Asthma** | 3 | 0.00 | 3 | 0.01 | 5 | 0.01 | 0.61 | 0.15 | 2.57 | 0.66 | 0.12 | 2.74 |
| **Lymphadenitis** | 7 | 0.01 | 4 | 0.01 | 4 | 0.01 | 1.79 | 0.52 | 6.11 | 1.09 | 0.27 | 4.36 |
| **Mumps** | 3 | 0.00 | 1 | 0.00 | 4 | 0.01 | 0.77 | 0.17 | 3.42 | 0.27 | 0.03 | 2.44 |
| **Ear periarthritis** | 2 | 0.00 | 5 | 0.01 | 4 | 0.01 | 0.51 | 0.09 | 2.79 | 1.36 | 0.37 | 5.08 |
| **Dermatitis** | 3 | 0.01 | 2 | 0.00 | 4 | 0.01 | 0.77 | 0.17 | 3.42 | 0.55 | 0.10 | 2.98 |
| Unknown | 487 | 1.00 | 254 | 0.46 | 374 | 0.60 | 1.33 | 1.16 | 1.52 | 0.74 | 0.63 | 0.87 |
| **Others** | 53 | 0.09 | 94 | 0.18 | 52 | 0.07 | 1.04 | 0.71 | 1.53 | 1.97 | 1.41 | 2.77 |

Note: data were clustered by kindergartens. Subscript 1indicates the intervention arm 1 vs. the control arm; subscript 2 indicates the intervention arm 2 vs. the control arm.

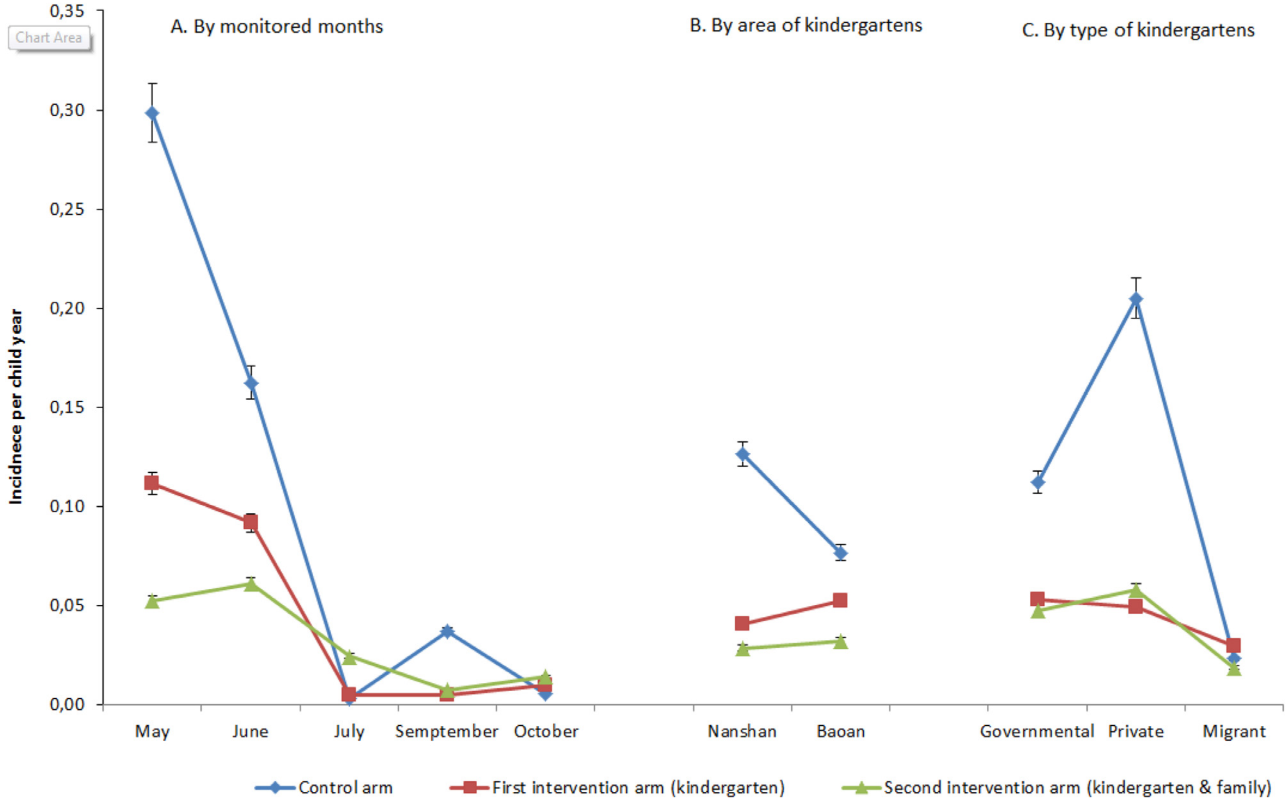


**Fig. 2.** Effect of the hand hygiene intervention on incidence of absent episodes of hand, foot and mouth disease among children attending kindergartens in Shenzhen, by follow-up month, area and type of kindergartens (year: 2015). *Note: all incidence rates were calculated with adjustment for clustering by kindergartens.*

Please cite this article as: X. Liu et al., A hand hygiene intervention to decrease hand, foot and mouth disease and absence due to sickness among kindergarteners in China: A cluster-randomized controlled trial, Journal of Infection (2018), https://doi.org/10.1016/j.jinf.2018.08.009

**Table 3**
Predicting count of absence due to hand, foot and mouth disease among children attending kindergartens in multilevel Poisson regression models.

| | Univariate | | | Multivariable | | |
|---|---|---|---|---|---|---|
| | IRR | 95%CI (IRR) | *P*-value | IRR | 95%CI (IRR) | *P*-value |
| **Intervention group** | | | | | | |
| Arm 1 | 0.39 | 0.26–0.59 | <0.001** | 0.48 | 0.17–1.32 | 0.155* |
| Arm 2 | 0.30 | 0.19–0.49 | <0.001** | 0.26 | 0.08–0.82 | 0.022** |
| Control | Ref. | | | | | |
| **Area of kindergartens** | | | | | | |
| Urban | 0.75 | 0.53–1.06 | 0.107* | 0.69 | 0.32–1.47 | 0.328 |
| Semi-urban | Ref. | | | | | |
| **Type of kindergartens** | | | | | | |
| Government | 3.23 | 1.89–5.51 | <0.001** | 3.14 | 1.18–8.38 | 0.023** |
| Private | 3.10 | 1.79–5.38 | <0.001** | 3.26 | 1.21–8.79 | 0.020** |
| Migrant | Ref. | | | | | |
| **Follow-up month** | | | | | | |
| October | 0.09 | 0.03–0.21 | <0.001** | 0.05 | 0.01–0.22 | <0.001** |
| September | 0.14 | 0.06–0.29 | <0.001** | 0.16 | 0.07–0.38 | <0.001** |
| July | 0.09 | 0.03–0.21 | <0.001** | 0.03 | 0.01–0.20 | <0.001** |
| June | 0.92 | 0.63–1.33 | 0.639 | 0.84 | 0.52–1.36 | 0.476 |
| May | Ref. | | | | | |

Note: * indicates $P < 0.20$; ** indicates $P < 0.05$. Intervention arm 1 involves the intervention delivered in kindergartens; Intervention arm 2 involves the intervention delivered in kindergartens and participated children's families; All models included log (child years at risk) as an offset and adjustment for clustering by kindergartens. Interaction of intervention group and follow-up month was included in the multivariable model.

in the second intervention arm had 0.26 times the incidence rate for HFMD (95% CI: 0.08–0.82), after correcting for the follow-up period, area and type of kindergartens in multilevel Poisson regression analyses (Table 3).

### *Effect of the trial on other diseases and the absence duration due to sickness*

We also monitored the effect of the intervention on other diseases (Table 2). The incidence of respiratory infections generally was 3.19 episodes per child-year in the first intervention arm; this was significantly lower than the incidence of 3.69 episodes in the control arm ($IRR_1$: 0.83, 95%CI: 0.79–0.88). The first intervention arm had lower incidences of Ophthalmitis ($IRR_1$: 0.41, 95%CI: 0.22–0.76) and Otitis media ($IRR_1$: 0.15, 95%CI: 0.03–0.64). In addition, both of the intervention arms had lower incidences of Varicella ($IRR_1$: 0.27, 95%CI: 0.10–0.72; $IRR_2$: 0.12, 95%CI: 0.03–0.49) and Measles ($IRR_1$: 0.24, 95%CI: 0.07–0.83; $IRR_2$: 0.09, 95%CI: 0.01–0.64). However, the second intervention arm had more reported episodes of absence due to gastrointestinal infections, and oral ulcer than the control arm ($IRR_2 > 1$; $P < 0.05$). No significant differences between the intervention arms and the control arm were found regarding the incidence of irritability, trauma, indigestion, saprodontia, eczema, asthma, lymphadenitis, mumps, ear periarthritis and dermatitis ($P > 0.05$).

On average, the duration of absence due to sickness in children attending kindergartens was 2.03 days (95%CI: 1.93–2.13) in the first intervention arm and 2.35 days (95%CI: 2.23–2.48) in the second intervention arm, which were significantly shorter than the duration of 2.65 days in the control arm (95%CI: 2.53–2.76). Controlling for the area, kindergarten type and grade level of children in multilevel regression analyses, on average, children in the control arm had 0.58 day (95%CI: 0.41–0.74) longer absences due to sickness than those in the first intervention arm, and 0.34 day (95%CI: 0.17–0.50) longer absence than those in the second intervention arm.

## Discussion

The HH intervention delivered in this study decreased the number of HFMD infections and reduced the duration of absence due to sickness in kindergartens in Shenzhen, China. By the 6-month follow-up, there had been fewer episodes of absence due to HFMD and shorter absence duration due to sickness in both intervention arms compared to the control arm. However, the difference between the two intervention arms was not statistically significant.

This is one of the first HH intervention studies to assess the impact of a HH intervention on HFMD infection among kindergarten children. The study applied a randomized controlled study design, there was a relatively large sample size of over 8000 children in 18 kindergartens, and the children had a high level of exposure to intervention components. Moreover, the study had a very high compliance of follow-up with regard to health monitoring, which was well integrated into the mandatory work routine of the kindergartens' teachers and school doctors. The HFMD incidence cases were reported mainly between May and June, as well as September, in this study, both which are consistent with the seasonal peaks identified by a recent publication using Shenzhen municipal pathogen surveillance data between 2008 and 2015.[25] Other strengths of the study are that data was collected on both episodes of absence and duration of absence for a comprehensive assessment of the intervention impact, and that the study covered many infectious diseases other than HFMD. The intervention in this study was developed based on a previous Dutch study on determinants of HH behaviour and composed multiple tailored components targeting the underlying determinants. The strategies and materials used were highly appraised by the participating kindergartens and families according to a focus-group study that we conducted at the first month follow-up (unpublished data). In addition, kindergartens in the control arm received the intervention after data collection, which perhaps also facilitated the recruitment of kindergartens and minimized loss to follow-up.

The study had several limitations though. First, the effect on disease incidence was not corrected for baseline due to lack of data. The kick-off activities which were conducted at the beginning of the intervention significantly increased the awareness and HH compliance of children and teachers in intervention arms. However, its creativity attracted a lot of attention from local media. As a result, it is likely that the intervention contaminated the control arm, and thus the incidence at the first month follow-up could

Please cite this article as: X. Liu et al., A hand hygiene intervention to decrease hand, foot and mouth disease and absence due to sickness among kindergarteners in China: A cluster-randomized controlled trial, Journal of Infection (2018), https://doi.org/10.1016/j.jinf.2018.08.009

not be used as the baseline. More intervention studies are needed with baseline measurements. Second, this trial involves only two districts in Shenzhen, which has a profile of having a relatively high socio-economic status and historically a lower prevalence of HFMD. Therefore, the findings might not apply to other kindergartens with different socio-economic backgrounds and prevalence of HFMD. Third, some of the diagnoses were self-reported by parents and thus are susceptible to information bias and social desirability bias.[26]. Third, although we delivered the intervention with a standard instruction for both intervention arms, at the follow-up we did not collect data regarding the exact consumption of HH products and educational materials. Therefore, the exact effect of each component of the intervention is unknown. Finally, we did not observe any significant difference in the incidences of measured infections between the two intervention arms due to the suboptimal involvement of the parents, and no information was collected on parents unwilling to participate in the intervention. It is common for parents of children attending kindergartens to work full time and therefore they may have been too busy to be involved.

The effect of this intervention in significantly decrease in the infection occurrence and absence duration due to illness might be mainly caused by the significant increase in the HH compliance of both teachers and children during the follow-up period, which we assessed in the same trial (unpublished yet), rather than a reduction in the severity or duration of the illness. Improved HH compliance can help prevent children from getting infections, specifically by preventing germs from getting into their eyes, nose, and mouth, as well as into their food and drinks and on other objects, like handrails, table tops, or toys, and then transferred to other children's hands. Besides HFMD, the intervention also decreased episodes of absence due to respiratory infections, ophthalmitis, otitis media, varicella and measles. Our findings are consistent with those of the previous study by Carabin and colleagues, which suggest that their intervention reduced the incidence of upper respiratory tract infections.[27] Moreover, similar to this study, our previous study using a similar intervention in the Netherlands did not find a reduction in diarrhoea,[14] and the results of a meta-analysis on the effect of HH in the community setting indicate that removing pathogens through HH may help prevent skin and eye infections.[28] However, there is little evidence available showing that HH interventions are associated with fewer cases of HFMD, and previous HH intervention studies generally show different effects from each other on infections and/or illness absenteeism.[29] More evidence is urgently needed to understand the importance of HH interventions in reducing disease occurrence in children.

In China the incidence of HFMD has continued to increase in recent years, and it is becoming a serious problem for children, their families and public health in general.[30,31] The results of this study show that a HH intervention delivered in both kindergartens and families can significantly reduce HFMD incidence, regardless of the season variation of HFMD and location and type of kindergartens. Therefore, it can provide a reference for the promotion of HH intervention and HFMD control in kindergartens to include family members of children. Compared to HFMD vaccination, HH interventions are cheaper, safer and easier to implement, so they are more suitable for improving children's health in developing countries.[32] Kindergarten is an ideal place for conducting HH interventions, as many children between the ages of three and six years are gathered in kindergarten classrooms, and teachers and school doctors can contribute to the implementation work. This makes it relatively easier to provide HH products, distribute health education materials and conduct team training sessions in kindergartens than in communities. However, HFMD cases in China occur more often among pre-school children, from zero to three years of age, who live scattered throughout different communities.[33] These children are more difficult to reach. Therefore, how to improve the HH intervention strategies in this study and apply them to the younger, scattered children in communities is the direction of our future research.

In conclusion, this study shows that our evidence-based HH intervention is effective at reducing HFMD infections and absence due to illness in children attending kindergartens in China. We recommend that future intervention studies consider our HH behaviour-change techniques to prevent infection in child-care settings and that they include family members of children to maximize the effect of those techniques in preventing HFMD infections. To scale up the impact of this intervention in the prevention of HFMD, a range of implementation research including cost-effective analyses will be our next step.

## Declaration of conflicting interests

The authors declare no potential conflicts of interests with respect to the research, authorship, and/or publication of this article. The hand hygiene products used in this study were jointly sponsored by Essity Hygiene Corporate and Vinda™ China Group; however, neither had any involvement during the study, including data collection, data analysis and preparation of the manuscript.

## Funding

The study was made possible with support from the Health and Family Planning Commission of Shenzhen Municipality in China, and the Department of Public Health, Erasmus University Medical Centre Rotterdam in the Netherlands. The funding source played no role in the study design, data analysis, or preparation of the manuscript.

## Ethical approval

This trial's protocol is registered and approved by the Ethics Committee of Shenzhen CDC (Registration no. 2015005) and the Netherlands Trial Registry (TC-5395). Informed consent to participate was collected from the parents and kindergarten doctors.

## Acknowledgements

We grateful acknowledge Daan Nieboer for valuable statistical advice. We thank the kindergartens and parents for their willingness to participate in the study, and the Nanshan District CDC and the Baoan District CDC for the assistance in the intervention and field investigation. Hand hygiene products used in this study were jointly sponsored by Essity Hygiene Corporate and Vinda™ China Group.

## References

1. WPRO. Hand, Foot and Mouth Disease. Available from: http://www.wpro.who.int/mediacentre/factsheets/fs_10072012_HFMD/en/.
2. Melnick J. *Enteroviruses: polioviruses, coxsackieviruses, echoviruses, and newer enteroviruses*. Fields DK BN, Howley PM, editors. 3rd ed.. Philadelphia: Lippincott-Raven; 1996.
3. Liu MY, Liu W, Luo J, Liu Y, Zhu Y, Berman H, et al. Characterization of an outbreak of hand, foot, and mouth disease in Nanchang, China in 2010. *PloS One* 2011;**6**(9):e25287 PubMed PMID: 21980416. Pubmed Central PMCID: 3182205. Epub 2011/10/08. eng.
4. Liankai Z, Chun C, Zongqiu C, Jianyun L, Lei L, Xingcai X. Risk factors of hand-foot-mouth disease in kindergartens in Guangzhou. *Chin J Sch Health* 2012;**33**(10):1214–16 chi.
5. Huang CC, Liu CC, Chang YC, Chen CY, Wang ST, Yeh TF. Neurologic complications in children with enterovirus 71 infection. *N Engl J Med* 1999;**341**(13):936–42 PubMed PMID: 10498488. Epub 1999/09/25. Eng.
6. Zhang Y, Zhu Z, Yang W, Ren J, Tan X, Wang Y, et al. An emerging recombinant human enterovirus 71 responsible for the 2008 outbreak of hand foot and mouth disease in Fuyang city of China. *Virol J* 2010;**7**:94 PubMed PMID: 20459851. Pubmed Central PMCID: Pmc2885340. Epub 2010/05/13. Eng.

7. Xing W, Liao Q, Viboud C, Zhang J, Sun J, Wu JT, et al. Hand, foot, and mouth disease in China, 2008-12: an epidemiological study. *Lancet Infect Dis* 2014;**14**(4):308–18 PubMed PMID: 24485991. Pubmed Central PMCID: Pmc4035015. Epub 2014/02/04. Eng.
8. Jin M, Xu X, Yuan L, Hanwu M, Jinquan C. Epidemiologic characteristics of hand-foot-mouth disease in Shenzhen City (2008-June, 2009). *J Public Health Prev Med* 2010;**21**(1):78–80.
9. Health CsMo. *Public health response for aggregation and outbreak of hand, foot and mouth disease*. Beijing, China: China's Ministry of Health; 2012.
10. Wang YR, Sun LL, Xiao WL, Chen LY, Wang XF, Pan DM. Epidemiology and clinical characteristics of hand foot, and mouth disease in a Shenzhen sentinel hospital from 2009 to 2011. *BMC Infect Dis* 2013;**13**:539 PubMed PMID: 24225231. Pubmed Central PMCID: 3829808. Epub 2013/11/15. eng.
11. Sanicas M. Severe hand, foot, and mouth disease (HFMD) is now vaccine-preventable (at least in China)! 2016.
12. WHO. Evidence for hand hygiene guidelines. 2016.
13. Xuan le TT, Rheinlander T, Hoat LN, Dalsgaard A, Konradsen F. Teaching handwashing with soap for schoolchildren in a multi-ethnic population in northern rural Vietnam. *Glob Health Action* 2013;**6**:1–12 PubMed PMID: 23618342. Pubmed Central PMCID: Pmc3636419. Epub 2013/04/27. eng.
14. Zomer TP, Erasmus V, Vlaar N, van Beeck EF, Tjon ATA, Richardus JH, et al. A hand hygiene intervention to decrease infections among children attending day care centers: design of a cluster randomized controlled trial. *BMC Infect Dis* 2013;**13**:259 PubMed PMID: 23731525. Pubmed Central PMCID: Pmc3673827. Epub 2013/06/05. eng.
15. Mei H, Xiurong L, Yuqing L, Yuan C, Yan C, Chun C. Study on hand washing behavior of pupils in Beijing. *Chin J Sch Health* 2013;**34**(6):670–1 4. chi.
16. Yue C, Shengxu Y, Yafei H, Fan H. Naturalistic observation on handwashing among students and teachers in primary and secondary schools of Taizhou. *J Chin J Sch Health* 2015;**36**(5):696–8 chi.
17. Zomer TP, Erasmus V, Looman CW, EF VANB, Tjon ATA, Richardus JH, et al. Improving hand hygiene compliance in child daycare centres: a randomized controlled trial. *Epidemiol Infect* 2016;**144**(12):2552–60 PubMed PMID: 27193613. Pubmed Central PMCID: Pmc4988269. Epub 2016/05/20. eng.
18. van Breukelen GJP, Candel MJJM. Calculating sample sizes for cluster randomized trials: we can keep it simple and efficient!. *J Clin Epidemiol* 2012;**65**(11):1212–18 PubMed PMID: WOS:000309571500011. English.
19. Correa JC, Pinto D, Salas LA, Camacho JC, Rondon M, Quintero J. A cluster-randomized controlled trial of handrubs for prevention of infectious diseases among children in Colombia. *Rev Panam Salud Publ* 2012;**31**(6):476–84 PubMed PMID: WOS:000314404800005. English.
20. Juanjuan T, Huazhi X, Huaxiu Y. The analysis of health status of children from government-sponsored kindergartens and prviate kindergartens in Rongcheng district. Matern Child Health Care China 2010;25(29):4242–43. Chinese.
21. Zomer TP, Erasmus V, Looman CW, Tjon ATA, Van Beeck EF, De Graaf JM, et al. A hand hygiene intervention to reduce infections in child daycare: a randomized controlled trial. *Epidemiol Infect* 2015;**143**(12):2494–502 PubMed PMID: 25566827. Pubmed Central PMCID: Pmc4531476. Epub 2015/01/09. Eng.
22. Kreuter MW, McClure SM. The role of culture in health communication. *Annu Rev Public Health* 2004;**25**:439–55 PubMed PMID: 15015929. Epub 2004/03/16. eng.
23. WHO. International Statistical Classification of Diseases and Related Health Problems 10th Revision (ICD-10). 2016.
24. Rothman KJ,SG. *Modern epidemiology*. 3rd ed. Philadelphia: Lippincott Williams & Wilkins; 2008.
25. Hong Y, Zhang Z, Long C, Yao XJ, Zhang HL, Xie X, et al. Epidemiology characteristics and pathogen surveillance of hand, foot and mouth disease in Shenzhen, 2008-2015. *Int J Virol* 2017;**24**(6):375–80.
26. Pan SC, Tien KL, Hung IC, Lin YJ, Sheng WH, Wang MJ, et al. Compliance of health care workers with hand hygiene practices: independent advantages of overt and covert observers. *PloS One* 2013;**8**(1):e53746 PubMed PMID: 23341991. Pubmed Central PMCID: Pmc3544847. Epub 2013/01/24. eng.
27. Carabin H, Gyorkos TW, Soto JC, Joseph L, Payment P, Collet JP. Effectiveness of a training program in reducing infections in toddlers attending day care centers. *Epidemiology* 1999;**10**(3):219–27 PubMed PMID: 10230828. Epub 1999/05/07. eng.
28. Aiello AE, Coulborn RM, Perez V, Larson EL. Effect of hand hygiene on infectious disease risk in the community setting: a meta-analysis. *Am J Public Health* 2008;**98**(8):1372–81 PubMed PMID: 18556606. Pubmed Central PMCID: Pmc2446461. Epub 2008/06/17. eng.
29. Huskins WC. Transmission and control of infections in out-of-home child care. *Pediatr Infect Dis J* 2000;**19**(10) S106-S10. PubMed PMID: WOS:000165070300004. English.
30. Zhao J, Li X. Determinants of the transmission variation of hand, foot and mouth disease in China. *PloS One* 2016;**11**(10):e0163789 PubMed PMID: 27701445. Epub 2016/10/05. Eng.
31. Guo C, Yang J, Guo Y, Ou QQ, Shen SQ, Ou CQ, et al. Short-term effects of meteorological factors on pediatric hand, foot, and mouth disease in Guangdong, China: a multi-city time-series analysis. *BMC Infect Dis* 2016;**16**(1):524 PubMed PMID: 27682137. Pubmed Central PMCID: Pmc5041518. Epub 2016/09/30. Eng.
32. Zhang D, Li Z, Zhang W, Guo P, Ma Z, Chen Q, et al. Hand-washing: the main strategy for avoiding hand, foot and mouth disease. *Int J Environ Res Public Health* 2016 Jun 18;**13**(6) PubMed PMID: 27322307. Pubmed Central PMCID: Pmc4924067. Epub 2016/06/21. Eng.
33. Xie YH, Chongsuvivatwong V, Tan Y, Tang Zh Z, Sornsrivichai V, McNeil EB. Important roles of public playgrounds in the transmission of hand, foot, and mouth disease. *Epidemiol Infect* 2015;**143**(7):1432–41 PubMed PMID: 25170900. Epub 2014/08/30. eng.

Please cite this article as: X. Liu et al., A hand hygiene intervention to decrease hand, foot and mouth disease and absence due to sickness among kindergarteners in China: A cluster-randomized controlled trial, Journal of Infection (2018), https://doi.org/10.1016/j.jinf.2018.08.009
